# Postural Control in Childhood: Investigating the Neurodevelopmental Gradient Hypothesis

**DOI:** 10.3390/ijerph18041693

**Published:** 2021-02-10

**Authors:** Leonardo Zoccante, Marco Luigi Ciceri, Liliya Chamitava, Gianfranco Di Gennaro, Lucia Cazzoletti, Maria Elisabetta Zanolin, Francesca Darra, Marco Colizzi

**Affiliations:** 1 Child and Adolescent Neuropsychiatry Unit, Maternal-Child Integrated Care Department, Integrated University Hospital of Verona, 37126 Verona, Italy; leonardo.zoccante@aovr.veneto.it (L.Z.); marco.ciceri@aulss9.veneto.it (M.L.C.); francesca.darra@univr.it (F.D.); 2 Unit of Epidemiology and Medical Statistics, Department of Diagnostics and Public Health, University of Verona, 37134 Verona, Italy; liliya.chamitava@univr.it (L.C.); lucia.cazzoletti@univr.it (L.C.); elisabetta.zanolin@univr.it (M.E.Z.); 3 Department of Pathology and Diagnostics, Integrated University Hospital of Verona, 37126 Verona, Italy; gianfradig@gmail.com; 4 Section of Psychiatry, Department of Neurosciences, Biomedicine and Movement Sciences, University of Verona, 37134 Verona, Italy; 5 Department of Psychosis Studies, Institute of Psychiatry, Psychology and Neuroscience, King’s College London, London SE5 8AF, UK

**Keywords:** autism spectrum disorder, attention deficit hyperactivity disorder, Tourette disorder, transdiagnostic approach, mental health prevention

## Abstract

Neurodevelopmental disorders (NDDs) have been suggested to lie on a gradient continuum, all resulting from common brain disturbances, but with different degrees of impairment severity. This case-control study aimed to assess postural stability against such hypothesis in 104 children/adolescents aged 5–17, of whom 81 had NDDs and 23 were healthy controls. Compared to healthy controls, Autism Spectrum Disorder (ASD) resulted in the most severely impaired neurodevelopmental condition, followed by Attention Deficit Hyperactive Disorder (ADHD) and Tourette Syndrome (TS). In particular, while ASD children/adolescents performed worse than healthy controls in a number of sensory conditions across all parameters, ADHD children/adolescents performed worse than healthy controls only in the sway area for the most complex sensory conditions, when their vision and somatosensory functions were both compromised, and performance in Tourette Syndrome (TS) was roughly indistinguishable from that of healthy controls. Finally, differences were also observed between clinical groups, with ASD children/adolescents, and to a much lesser extent ADHD children/adolescents, performing worse than TS children/adolescents, especially when sensory systems were not operationally accurate. Evidence from this study indicates that poor postural control may be a useful biomarker for risk assessment during neurodevelopment, in line with predictions from the gradient hypothesis.

## 1. Introduction

Since the 1960s, a rising prevalence of childhood disabilities has been documented, largely because of an increase in the prevalence of mental and behavioral conditions such as Autism Spectrum Disorder (ASD), Attention Deficit Hyperactive Disorder (ADHD), and Tourette Syndrome (TS), whereas the prevalence of any other developmental delay such as cerebral palsy, hearing loss, and seizures, declined over time [1]. ASD, ADHD, and TS share common symptomatic features [2], such as impairments in the fields of general development [3], communication and language [3,4], social inter-relatedness [3,5,6,7], motor coordination [8], attention [9], activity [10,11], behavior, mood [7,12,13], and sleep [7,14,15,16]. However, it is also important to recognize that although symptoms may overlap, this does not always imply the same presentation of symptoms or the same response to treatment efforts. Thus, from a phenotypic perspective, similar behavioral manifestations may exist across conditions and similar behaviors may manifest differently within a condition [17]. In light of such extensive difficulties, these conditions can affect individuals’ personal (e.g., impaired executive functions), social, and school skills (e.g., impaired learning), with implications for future working abilities [2]. This is of paramount importance, considering that interpersonal skills may per se be less proficient among individuals with neurodevelopmental conditions, making it difficult to infer directionality of effects. It is plausible that individuals with neurodevelopmental conditions may be differentially susceptible to a variety of environmental factors with negative effects on their preexisting behavioral difficulties [18].

Despite evidence of comorbidity [5,10,19], ASD, ADHD, and TS were previously considered different from each other [20]. Only recently have they been grouped into the single diagnostic category of neurodevelopmental disorders (NDDs) [3] because of substantial overlapping not only at the clinical [5,6,21,22] but also at the neurobiological level [23,24,25,26,27]. This has led to the hypothesis that NDDs, including those that typically emerge in late adolescence and early adulthood such as affective and non-affective psychoses, should be seen as lying on an etiological and neurodevelopmental gradient continuum, all resulting from the commonality of disrupted or deviant brain development [28], but with different degrees of neurodevelopmental impairment severity [20]. According to the neurodevelopmental gradient hypothesis, the earlier the age of onset and the higher the severity and persistence of the psychopathological, cognitive, genetic, and sensorimotor impairment, the greater the overall neurodevelopmental impairment [20]. In line with this, when such impairments are compared across disorders, the rates are in decreasing severity from ASD to late-onset NDD [20].

Motor abnormalities are core features of ASD (e.g., stereotypic movements), ADHD (e.g., hyperactivity), and TS (e.g., tics), and have been suggested to represent a trans-diagnostic domain putatively sharing neurobiological mechanisms of neurodevelopmental origin [29]. Motor difficulties, especially in the coordination domain, have also been reported in typically developing children, potentially reflecting age-dependent reversible developmental traits [30]. However, their persistence in late childhood is suggestive of a disrupted sensory integration, thus affecting the sequencing of complex motor acts [31,32], and seem to be related with poor cognitive performance [8] in predicting the manifestation of an NDD [8,29]. Further, a developmental coordination disorder, the most severe phenotype of coordination impairment, once defined as “dyspraxia” or “motor clumsiness”, is frequently diagnosed in children with an NDD [33,34,35].

Abnormal sensory responsivity has been implicated in atypical neurodevelopment, independently of concomitant motor difficulties [36]. Research evidence indicates that sensory feedback and movement are intrinsically connected [37], as a variety of sensory information from the environment needs to be integrated in order to plan and execute movement effectively [36]. Studies conducted over the last decade have started to explore the contribution of aberrant sensorimotor integration, defined as an impairment in the pathway involving motor activity triggered by sensory stimuli, to the development and maintenance of NDDs [38].

Sensorimotor integration deficits among individuals with neurodevelopmental [39,40] and other developmental [41] conditions may affect postural control in both static and dynamic conditions. Standing balance requires the ability to integrate sensory inputs from visual, somatosensory, and vestibular systems [42]. Briefly, as we interact with our environment, the central vestibular system receives regular afferent fibers transmitting detailed information about head rotations through precise spike-timing as well as irregular afferents responding to high-frequency features exclusively through changes in the firing rate. Then, the brain combines vestibular and extra-vestibular cues, such as visual and proprioceptive information, at the earliest stages of central vestibular processing to construct an estimate of self-motion. Finally, vestibular processing is shaped as a function of context during reflex behavior as well as more complex voluntary behaviors. Thus, disturbances in the multisensory integration by the brain may disrupt the accurate control of behavior in everyday life, including posture and balance [43]. Both preclinical and clinical studies converge on the evidence that efficient multisensory integration depends on intact feedback and feedforward neuronal loops between cortical regions, including primary sensory regions as well as multisensory areas such as the superior temporal sulcus and motor regions, and subcortical regions such as the thalamus. These cortico-cortical and cortico-subcortical transmissions have been suggested to serve a central integrative mechanism where visual, somatosensory, and vestibular inputs converge to support postural stability [44].

Experimental perturbation of sensory inputs can help in examining how individuals suffering from different conditions utilize combinations of that sensory feedback to maintain an upright stance [45]. In line with the National Institute of Mental Health (NIMH) Research Domain Criteria (RDoC) project, which promotes a framework for translational research on functional neurobehavioral dimensions across different disorder categories, sensorimotor systems may well represent a domain of function to be studied in neurodevelopment [46]. However, although previous studies have examined balance performances in developmental disorders [39,40,41], sensorimotor integration processes across different NDDs have not been systematically assessed. The present study attempted to fill this gap by performing a case-control analysis of postural stability under normal and altered sensory conditions in NDDs (ASD, ADHD, and TS) as compared to healthy controls. We hypothesized that, compared to healthy controls, children/adolescents with an NDD would present with decreasing postural balance impairment from ASD to ADHD and TS, in line with predictions from the gradient hypothesis.

## 2. Materials and Methods

### 2.1. Participants

Volunteers were enrolled in a case-control study through convenience sampling, based on their willingness to participate, at the Veneto Autism Spectrum Disorder Regional Centre, Integrated University Hospital of Verona, Italy. Participants aged 5 to 17 were assessed for the presence of a neurodevelopmental disorder (NDD) and recruited if they fulfilled the Diagnostic and Statistical Manual of Mental Disorders, fifth edition, (DSM-5) criteria for one of the following conditions: (a) Autism Spectrum Disorder (ASD), (b) Attention Deficit Hyperactivity Disorder (ADHD), (c) Tourette Syndrome (TS). Patients were excluded if presenting with (a) a formal comorbid neurodevelopmental condition, i.e., satisfying DSM-5 diagnostic criteria for more than one neurodevelopmental condition (e.g., receiving diagnosis of both ASD and TS); (b) a formal comorbid neuropsychiatric condition, i.e., satisfying DSM-5 diagnostic criteria for another neuropsychiatric condition such as psychosis-related disorders, depression-related disorders, anxiety-related disorders, and obsessive–compulsive-related disorders (e.g., receiving diagnosis of both ADHD and major depressive disorder); (c) a clinically relevant medical condition, particularly a neurological (receiving diagnosis of cerebral palsy, epilepsy, or otherwise-classified motor handicap) or orthopedic (receiving diagnosis of fracture or severe injury) condition; (d) a genetic syndrome (receiving diagnosis of chromosomal abnormalities); (e) a severe form of atypical neurodevelopment rendering it difficult to satisfactorily perform the study (all ASD children/adolescents included in the study had a diagnosis ranked severity level 1, which is the least severe form in terms of needed support, according to the DSM-5 three-level severity classification). Such exclusion criteria were applied in order to reduce the implications of “spurious comorbidity”, which is the higher co-occurrence of disorders in clinically ascertained samples than in population-based samples, possibly due to such patients presenting with comorbid conditions being more likely to seek medical care and receive a diagnostic evaluation.

Healthy peers were recruited outside of the hospital facility and enrolled into the study with the support of several primary and secondary schools and the Hospital Pediatric Unit of Verona. Children/adolescents who wanted to participate in the study were recruited only if presenting with good overall health. They were excluded if presenting with (a) a neurodevelopmental condition; (b) a neuropsychiatric condition; (c) a clinically relevant medical condition; (d) a genetic syndrome.

### 2.2. General Assessment

Socio-demographic information, such as age and gender, were obtained from all study participants. All volunteers were extensively visited by expert clinicians. Assessments included: (a) Review of clinical records, (b) in-depth physical exam, (c) medical history, (d) rating scales. Socio-demographic and clinical characteristics of the study sample, including cognitive performance as well as developmental motor and coordination abilities have been extensively described before [8]. Briefly, children/adolescents with an NDD presented with a lower range intelligence quotient, less proficient movement skills when compared with healthy peers’ normative data, and coordination performance indicative of potential developmental coordination difficulties [8]. The present report focuses on stabilometric data.

### 2.3. Postural Control

In order to assess postural control, all participants underwent stabilometry, the methods of which have already been reported in detail [45]. Briefly, stabilometric assessments were performed in a standing position on an electronic monoaxial platform known as the TecnoBody^®^ Platform (PK200WL, Prokin Tecnobody, Dalmine (BG), Italy). Participants’ age, height, and weight were entered into the software in order for results to be consistent with such anthropometric information. The placement of each participants’ feet on the platform was standardized with the medial malleolus at the rotation axis, as indicated by a V-shape, keeping a distance of 3 cm between the two malleoli, and extra-rotating 12° the medial borders of the feet.

**Figure 1:**
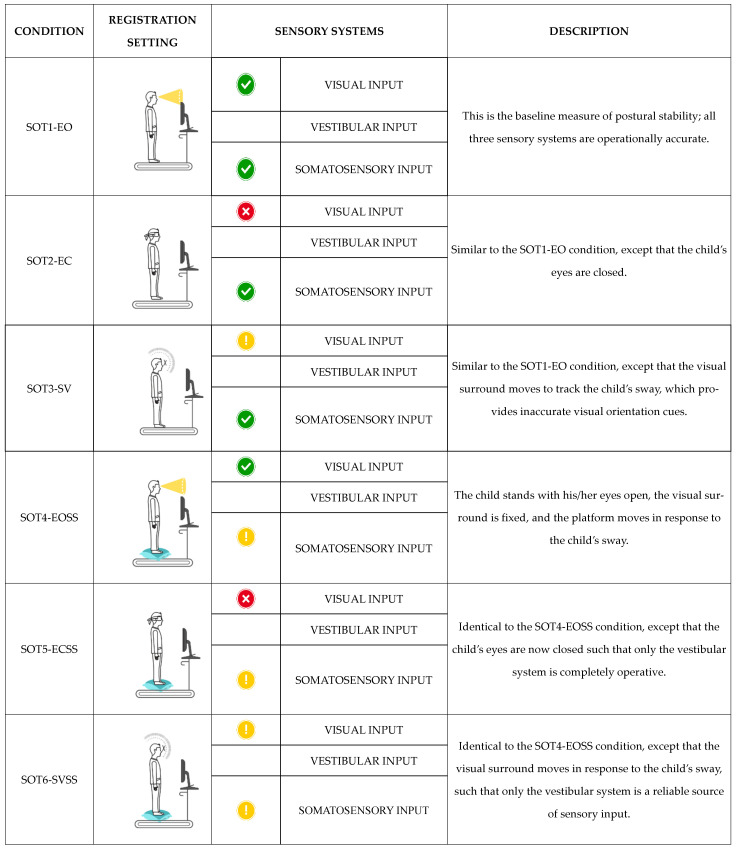
SOT: Sensory Organization Test; EO: Eyes Open; EC: Eyes Closed; SV: Sway-referenced Vision; EOSS: Eyes Open Sway-referenced Support; ECSS: Eyes Closed Sway-referenced Support; SVSS: Sway-referenced Vision Sway-referenced Support. Visual input is the sensation of any change in the visual environment. Vestibular input is the sensation of any change in position, direction, or movement of the head. Somatosensory input is the sensation of any change in, but not limited to, touch, temperature, posture, and limb position. 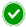 Normal sensory input; 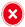 absent sensory input; 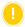 sway-referenced input.

Stabilometric performance was evaluated according to the Sensory Organization Test (SOT) [47], a protocol whose reliability and validity have been well established, also in pediatric populations with NDDs [48,49,50,51]. The SOT protocol allows quantifying subjects’ ability to effectively use visual, vestibular, and proprioceptive inputs, as well as suppress inexact sensory information while standing. It consists of six sensory conditions: (i) Eyes open and with fixed support (SOT1-EO); (ii) eyes closed and with fixed support (SOT2-EC); (iii) sway-referenced vision and with fixed support (SOT3-SV); (iv) eyes open and with sway-referenced support (SOT4-EOSS); (v) eyes closed and with sway-referenced support (SOT5-ECSS); and (vi) sway-referenced vision and sway-referenced support (SOT6-SVSS) [52] (Figure 1). For each condition, four distinct parameters were measured to describe postural control: (i) The sway area (mm^2^; area), that is the space covered due to body oscillations during the test; (ii) the length of the Center of Pressure (CoP) trajectory (mm; perimeter), that is the length of the route recorded due to body oscillations during the test; (iii) the mean velocity of the CoP displacement in the anteroposterior direction (mm/s; Anterior–Posterior Average Velocity (APAV)); and (iv) the mean velocity of the CoP displacement in the mediolateral direction (mm/s; Lateral Average Velocity (LAV)).

Two practice trials for each condition were conducted before the recording began. The test protocol consisted of three trials of each condition. Children/adolescents stood for 30 s for each condition.

### 2.4. Statistical Analyses

The descriptive statistics were presented as means and standard deviations (SD) for normally distributed continuous variables, and as medians and interquartile ranges (IQR) for continuous variables that failed the normality test (Shapiro–Wilk tests). Frequencies and percentages were used to describe categorical variables. To take into account the non-normal distributions of the data, for each of the four outcomes (area, perimeter, APAV, and LAV) and each of the six sensory conditions (SOT1-EO, SOT2-EC, SOT3-SV, SOT4-EOSS, SOT5-ECSS, and SOT6-SVSS), the comparison between cases and controls was performed through quantile regression models adjusted for gender and age. The threshold level selected for statistical significance was *p* < 0.05. All pairwise comparisons of adjusted medians were conducted and Bonferroni correction was applied to account for multiple testing. All calculated probabilities are presented as adjusted *p*-value after Bonferroni correction. The statistical analyses were performed with the statistical software Stata 16.1 (https://www.stata.com (accessed on 9 February 2021)).

### 2.5. Ethics

The research ethics committee at the Integrated University Hospital of Verona approved all protocols and procedures which led to the current study (CESC 2242 and CESC 2243). Parents and guardians of all study participants were offered an extensive description of the study and then consented to their inclusion in the study by signing an informed written consent. Consent was also obtained with reference to the publication of the collected research data.

## 3. Results

### 3.1. Socio-Demographic Information and Clinical Characteristics

Data were obtained on 104 participants, 81 of whom had a neurodevelopmental disorder (NDD) and 23 were healthy peers. As expected, there was a male-biased representation among children/adolescents with NDDs. Descriptive statistics of the study participants are reported in Table 1.

### 3.2. Postural Control

Medians of raw data for NDD as a whole group (ASD+ADHD+TS) and for each neurodevelopmental condition as well as for healthy controls are reported in Supplementary Tables S1–S5.

#### 3.2.1. Area

Medians adjusted for gender and age for the four groups (ASD, ADHD, TS, and healthy controls) are reported in Table 2. A graphical representation of such data is also presented in Supplementary Figure S1. After Bonferroni correction for multiple comparisons, there were statistically significant differences in the median area for the SOT1-EO (*p* = 0.025) and SOT6-SVSS (*p* = 0.003) conditions as well as a difference approaching significance for the SOT2-EC condition (*p* = 0.057) between ASD children/adolescents and healthy controls. Moreover, the median area for the SOT6-SVSS condition was significantly larger in ADHD children/adolescents as compared to healthy controls (*p* = 0.009).

Further, there was a difference approaching significance in the median area for the SOT1-EO condition (*p* = 0.099) as well as statistically significant differences for the SOT2-EC (*p* = 0.023) and SOT6-SVSS (*p* = 0.037) conditions between ASD children/adolescents and TS children/adolescents. Finally, there was a difference approaching significance in the median area for the SOT6-SVSS condition between ADHD and TS children/adolescents (*p* = 0.090).

#### 3.2.2. Perimeter

Medians adjusted for gender and age for the four groups (ASD, ADHD, TS, and healthy controls) are reported in Table 3. A graphical representation of such data is also presented in Supplementary Figure S2. After Bonferroni correction for multiple comparisons, the median Perimeter for the SOT2-EC condition was significantly longer in ASD children/adolescents as compared to healthy controls (*p* = 0.017).

Further, there was a difference approaching significance in the median Perimeter for the SOT2-EC condition between ASD and TS children/adolescents (*p* = 0.078).

#### 3.2.3. Anterior–Posterior Average Velocity

Medians adjusted for gender and age for the four groups (ASD, ADHD, TS, and healthy controls) are reported in Table 4. A graphical representation of such data is also presented in Supplementary Figure S3. After Bonferroni correction for multiple comparisons, the median Anterior–Posterior Average Velocity (APAV) for the SOT2-EC condition was significantly higher in ASD children/adolescents as compared to healthy controls (*p* = 0.003).

Further, there were differences approaching the significance in the median APAV for the SOT1-EO (*p* = 0.065) and SOT2-EC conditions (*p* = 0.086) between ASD and ADHD children/adolescents. Moreover, there were statistically significant differences for the SOT2-EC (*p* = 0.007) and SOT5-ECSS (*p* = 0.041) conditions between ASD children/adolescents and TS children/adolescents. Finally, there was a difference approaching significance in the median APAV for the SOT5-ECSS condition between ADHD and TS children/adolescents (*p* = 0.061).

#### 3.2.4. Lateral Average Velocity

Medians adjusted for gender and age for the four groups (ASD, ADHD, TS, and healthy controls) are reported in Table 5. A graphical representation of such data is also presented in Supplementary Figure S4. After Bonferroni correction for multiple comparisons, the median Lateral Average Velocity (LAV) for the SOT1-EO (*p* = 0.019) and SOT2-EC (*p* = 0.036) conditions was significantly higher in ASD children/adolescents as compared to healthy controls.

Further, the median LAV for the SOT2-EC condition was significantly higher in ASD children/adolescents as compared to TS children/adolescents (*p* = 0.013) as well as in ADHD children/adolescents as compared to TS children/adolescents (*p* = 0.042).

## 4. Discussion

To the best of our knowledge, this is the first case-control study to examine whether children/adolescents with neurodevelopmental disorders (NDDs) and healthy controls differ in terms of postural control, also examining whether NDD children/adolescents present with a different degree of impairment depending on the specific neurodevelopmental condition, in line with the neurodevelopmental gradient hypothesis. Results indicate that, as for any other impairment observed in atypical neurodevelopment [20], postural instability severity could be seen as lying on a neurodevelopmental gradient continuum, with decreasing severity from Autism Spectrum Disorder (ASD) to late-onset NDD. More specifically, four patterns of postural balance were observed in this study. First, ASD children/adolescents performed worse than healthy controls in a number of sensory conditions across all parameters. Second, Attention Deficit Hyperactive Disorder (ADHD) children/adolescents performed worse than healthy controls only for the most complex sensory condition (SOT6-SVSS) in the area parameter, when their vision and somatosensory functions were both compromised. Third, differences between Tourette syndrome (TS) children/adolescents and healthy controls in the performance across all parameters and conditions investigated failed to reach statistical significance. Fourth, differences were also observed between clinical groups. Specifically, ASD children/adolescents performed worse than TS children/adolescents in a number of conditions across all parameters, especially when sensory systems were not operationally accurate. To a much lesser extent, when receiving inaccurate sensory orientation cues, ADHD children/adolescents also tended to perform or performed worse than TS children/adolescents. Finally, ASD children/adolescents tended to perform worse than ADHD children/adolescents during the baseline condition (SOT1-EO) and when the visual input was absent (SOT2-EC) for the Anterior–Posterior Average Velocity (APAV) parameter. Therefore, in terms of postural control, ASD resulted in the most severely impaired neurodevelopmental condition, followed by ADHD and TS.

To date, while neuromotor symptoms are recognized as a core feature of most neurodevelopmental conditions, from those with childhood onset (e.g., stereotypic movements in ASD) to disorders with early adulthood onset (e.g., catatonia in psychosis) [3], normative motor development throughout a child’s early life is not clearly defined [53]. As a consequence, it is still not completely clear when to consider motor difficulties of pathological relevance rather than part of the child’s physiological brain maturation [53,54]. Moreover, at a research level, focusing on predefined motor characteristics of atypical neurodevelopment (e.g., stereotypic movements for ASD, hyperactivity for ADHD, tics for TS) [29] has offered limited support to our ability to differentiate pathognomonic from non-specific or benign motor phenomena [55]. Most research evidence agrees that motor difficulties in childhood do not necessarily imply a neuropsychiatric disorder, especially if not corroborated by additional evidence of brain lesions or abnormalities [30]. However, if such motor difficulties do persist in late childhood, they may require clinical attention as a potential sentinel of an underlying maturational delay, with implications for the child’s ability to integrate sensorimotor stimuli to perform complex motor acts, including maintaining postural balance [31,32].

A further level of complexity affecting our ability to completely understand neuromotor functioning in the context of neuropsychiatric conditions is reflected in the debate whether poor sensorimotor integration would be specific to psychosis symptom formation, as historically assumed, or independent of such diagnosis [56,57]. More recent research evidence of psychomotor dysfunction in major psychiatric conditions including depression [58,59], anxiety [60], and schizophrenia [61,62], seems to support the hypothesis that such dysfunction might reflect a generalized deficit of neural integration, which is not related to a single condition. However, psychomotor dysfunction would present with specific characteristics to each condition, possibly depending on the severity, timing, and predominant pattern of brain aberrances and resulting neuropsychological manifestations.

The conceptualization of NDDs in a neurodevelopmental continuum results from evidence for pleiotropy between ASD, ADHD, and TS, which refers to shared neurobiological risk explaining correlations among NDDs [63,64]. Findings from the present study are in line with such conceptual framework and seem to point toward a trans-diagnostic role of poor sensorimotor integration in atypical neurodevelopment, rather than lying on the discrete etiological pathway to a specific neurodevelopmental condition. Further, in line with the neurodevelopmental gradient hypothesis [20], poor postural control may be a useful biomarker for patient stratification across diagnostic boundaries in neurodevelopment, where the higher the sensorimotor severity, the greater the neurodevelopmental impairment.

Converging research evidence indicates that individuals with NDD present with both functional and structural brain abnormalities [65,66,67,68,69,70]. Interestingly, more limited evidence suggests both shared [71] and disorder-specific [72] structural brain alterations that vary over time and differently depending on the specific neurodevelopmental condition [73]. Distinct functional abnormalities have also been described, being more pronounced in ASD compared to other NDDs [74]. Further, differential alterations in functional connectivity between primary and supplementary motor cortex, and regions involved in brain motor circuitry such as putamen, thalamus, and cerebellum, have been suggested depending on the severity of the clinical presentation of atypical neurodevelopment [75]. Such evidence raises the question of a possible resulting impairment in the process of sensorimotor integration, that is the brain process allowing, by complex neural operations, the connection of the sensory and motor domains [76]. Deficiencies in sensorimotor integration would then present as difficulties in effectively utilizing sensory feedback to correct movements, resulting in the coordination difficulties and sensory reactivity abnormalities phenotypically observed among individuals with an NDD [77]. Altogether, the findings suggest a combination of shared and age-specific patterns of brain abnormalities reflecting overlapping and unique symptom presentations occupying a gradient of neurodevelopmental impairment.

While results seem to suggest that the impairment in sensorimotor integration can be graded according to the severity of the neurodevelopmental impairment putatively attributed to each NDD, with ASD being the most severe condition, we must beware of the risk of oversimplifying the diagnostic conundrum. Future studies will need to improve our understanding of the mechanisms underlying the co-occurrence of symptomatic manifestations such as deficits in sensorimotor integration among individuals with neurodevelopmental conditions. Such knowledge acquisition will help disentangle whether deficits in sensorimotor integration among NDDs reflect distinct dysfunctions or could imply different degrees of impairment on a common underlying neurodevelopmental continuum. Moreover, whether the degree of impairment in sensorimotor integration can make clear predictions about the outcome of NDDs, in line with the neurodevelopmental gradient hypothesis, is currently unclear. In addition, while motor impairments are described in both ASD and ADHD, evidence for specificity of motor impairment within different NDDs remains unclear. For instance, some studies indicate specific ASD-associated impairments in tasks requiring rapid integration of visual feedback, suggesting that individuals with ASD are less likely to rely on visual feedback when learning a novel movement pattern, instead showing a bias towards reliance on proprioceptive feedback [78,79]. In contrast, neither individuals with ADHD nor TS would present with such atypical bias in sensory-motor integration. Further, poor performance on manual dexterity tasks would be more strongly related to ADHD group membership, possibly as a consequence of ADHD-associated inefficient response selection negatively impacting motor control [80].

Results presented here need be seen in the light of some strengths and limitations. On the one hand, strengths include the implementation of rigorous inclusion criteria for each diagnostic group, as well as the investigation of postural control by instrumental assessment. As a consequence, the study design allows excluding that the observed postural instability would be due to the copresence of other physical of neuropsychiatric conditions or a poorly reliable postural assessment. On the other, the main limitations of the current research are that the sample size and gender imbalance did not allow to fully investigate the contribution of age and gender in the manifestation of NDD-related postural instability, though controlling for the confounding effects of such variables. Moreover, the study design did not contemplate a longitudinal evaluation of the phenomenon, thus requiring prospective studies to assess the evolution of postural control over time. Finally, future studies will need to explore the impact of sensorimotor difficulties on the development and maintenance of NDD core symptom severity as well as specific predominant subtype (e.g., ASD severity level, ADHD inattentive/hyperactive–impulsive presentation, TS motor/vocal tic presentation).

## 5. Conclusions

In conclusion, while requiring replication in larger samples, evidence from this study indicates that poor postural control may be a useful biomarker for risk assessment in individuals suspected of having an atypical neurodevelopment. Moreover, such impairment seems to answer to the neurodevelopmental gradient hypothesis, with autism spectrum disorder children/adolescents presenting with the most severe postural instability, followed by children/adolescents with attention deficit hyperactive disorder and Tourette syndrome. Altogether, findings from this study add to the growing evidence stressing the importance of orienting public-health decisions in the direction of improving atypical neurodevelopment detection by also including the evaluation of sensorimotor skills. In the presence of such difficulties, interventions aimed at enhancing motor abilities should be supported along with preexisting therapies targeting psychological, behavioral, and cognitive difficulties.

## Figures and Tables

**Table 1:** Socio-demographic and clinical characteristics of the study participants.

	ASD	ADHD	TS	Healthy Controls
N (%)				
Participants	20 (19.2)	31 (29.8)	30 (28.9)	23 (22.1)
Gender (male)	16 (80.0)	26 (83.9)	27 (90.0)	10 (43.5)
M (SD)				
Age (years) [range]	10.7 (2.0) [7.2–14.6]	10.0 (2.4) [5.7–15]	10.4 (2.2) [7.4–16]	12.3 (2.6) [8.3–16.9]
Total IQ [range]	94.1 (15.9) ^ [65–115]	97.0 (16.5) [60–141]	93.9 (16.7) ^^ [45–120]	105.2 (15.5) ^ [65–132]

ASD, Autism Spectrum Disorder; ADHD, Attention Deficit Hyperactivity Disorder; TS, Tourette Syndrome; M, mean; SD, standard deviation; IQ, Intelligence Quotient; ^, 1 missing value; ^^, 2 missing values.

**Table 2:** Performance in the area parameter among neurodevelopmental disorder (NDD) conditions and controls.

Conditions	ASD	ADHD	TS	Controls	
Adjusted Median * (95% CI)				
SOT1-EO	115 (82–147)	80 (53–106)	63 (36–90)	44 (11–78)	
SOT2-EC	234 (163–306)	148 (89–207)	94 (34–154)	93 (18–168)	
SOT3-SV	204 (130–279)	202 (141–264)	143 (80–205)	85 (7–163)	
SOT4-EOSS	148 (92–204)	132 (86–178)	118 (71–165)	90 (31–149)	
SOT5-ECSS	423 (288–559)	429 (318–541)	268 (156–381)	232 (90–373)	
SOT6-SVSS	573 (442–704)	521 (413–629)	331 (222–440)	216 (79–354)	

NDD, Neurodevelopmental Disorder; ASD, Autism Spectrum Disorder; ADHD, Attention Deficit Hyperactivity Disorder; TS, Tourette Syndrome; *, adjusted for gender and age; CI, Confidence Interval; SOT, Sensory Organization Test; EO, Eyes Open; EC, Eyes Closed; SV, Sway-referenced Vision; EOSS, Eyes Open Sway-referenced Support; ECSS, Eyes Closed Sway-referenced Support; SVSS, Sway-referenced Vision Sway-referenced Support.

**Table 3:** Performance in the perimeter parameter among NDD conditions and controls.

Conditions	ASD	ADHD	TS	Controls	
Adjusted Median * (95% CI)					
SOT1-EO	178 (152–204)	146 (125–167)	145 (123–166)	134 (107–161)	
SOT2-EC	276 (234–318)	224 (190–258)	207 (172–241)	181 (137–225)	
SOT3-SV	240 (196–283)	215 (179–251)	178 (142–215)	181 (135–227)	
SOT4-EOSS	236 (199–273)	245 (214–275)	207 (176–238)	219 (180–258)	
SOT5-ECSS	433 (369–496)	426 (374–478)	351 (298–403)	359 (293–425)	
SOT6-SVSS	429 (352–506)	392 (328–455)	339 (275–404)	354 (273–435)	

NDD, Neurodevelopmental Disorder; ASD, Autism Spectrum Disorder; ADHD, Attention Deficit Hyperactivity Disorder; TS, Tourette Syndrome; *, adjusted for gender and age; CI, Confidence Interval; SOT, Sensory Organization Test; EO, Eyes Open; EC, Eyes Closed; SV, Sway-referenced Vision; EOSS, Eyes Open Sway-referenced Support; ECSS, Eyes Closed Sway-referenced Support; SVSS, Sway-referenced Vision Sway-referenced Support.

**Table 4:** Performance in the Anterior–Posterior Average Velocity (APAV) parameter among NDD conditions and controls.

Conditions	ASD	ADHD	TS	Controls
Adjusted Median * (95% CI)				
SOT1-EO	5 (4–6)	4 (3–5)	4 (3–5)	4 (4–5)
SOT2-EC	9 (7–10)	7 (5–8)	6 (5–7)	5 (4–7)
SOT3-SV	6 (5–8)	6 (5–7)	5 (4–6)	5 (4–6)
SOT4-EOSS	6 (5–7)	7 (6–8)	5 (4–6)	6 (5–7)
SOT5-ECSS	12 (11–14)	12 (10–13)	9 (8–11)	11 (9–12)
SOT6-SVSS	11 (9–13)	11 (10–13)	9 (7–10)	10 (8–12)

APAV, Anterior–Posterior Average Velocity; NDD, Neurodevelopmental Disorder; ASD, Autism Spectrum Disorder; ADHD, Attention Deficit Hyperactivity Disorder; TS, Tourette Syndrome; *, adjusted for gender and age; CI, Confidence Interval; SOT, Sensory Organization Test; EO, Eyes Open; EC, Eyes Closed; SV, Sway-referenced Vision; EOSS, Eyes Open Sway-referenced Support; ECSS, Eyes Closed Sway-referenced Support; SVSS, Sway-referenced Vision Sway-referenced Support.

**Table 5:** Performance in the Lateral Average Velocity (LAV) parameter among NDD conditions and controls.

Conditions	ASD	ADHD	TS	Controls
Adjusted Median * (95% CI)				
SOT1-EO	4 (4–5)	3 (3–4)	3 (3–4)	3 (2–3)
SOT2-EC	6 (5–6)	5 (5–6)	4 (3–5)	4 (3–5)
SOT3-SV	5 (4–6)	5 (4–6)	4 (3–4)	4 (3–4)
SOT4-EOSS	5 (5–6)	5 (5–6)	5 (5–6)	5 (4–6)
SOT5-ECSS	10 (8–11)	10 (8–11)	8 (7–10)	9 (7–10)
SOT6-SVSS	10 (8–12)	9 (8–11)	8 (6–9)	7 (6–9)

LAV, Lateral Average Velocity; NDD, Neurodevelopmental Disorder; ASD, Autism Spectrum Disorder; ADHD, Attention Deficit Hyperactivity Disorder; TS, Tourette Syndrome; *, adjusted for gender and age; CI, Confidence Interval; SOT, Sensory Organization Test; EO, Eyes Open; EC, Eyes Closed; SV, Sway-referenced Vision; EOSS, Eyes Open Sway-referenced Support; ECSS, Eyes Closed Sway-referenced Support; SVSS, Sway-referenced Vision Sway-referenced Support.

## Data Availability

The data presented in this study are available on request from the corresponding author.

## Supplementary Materials

The following are available online at https://www.mdpi.com/1660-4601/18/4/1693/s1, Table S1: Performance among NDD children/adolescents as a whole group and controls; Table S2: Performance in the Area parameter among NDD and controls; Table S3: Performance in the Perimeter parameter among NDD and controls; Table S4: Performance in the APAV parameter among NDD and controls; Table S5: Performance in the LAV parameter among NDD and controls; Figure S1: The figure shows performance in the Area parameter among neurodevelopmental disorders and controls and bars show medians adjusted for gender and age; Figure S2. The figure shows performance in the Perimeter parameter among neurodevelopmental disorders and controls and bars show medians adjusted for gender and age; Figure S3. The figure shows performance in the Anterior-Posterior Average Velocity parameter among neurodevelopmental disorders and controls and bars show medians adjusted for gender and age; Figure S4. The figure shows performance in the Lateral Average Velocity parameter among neurodevelopmental disorders and controls and bars show medians adjusted for gender and age.
