# Supplementary material for: Postural Control in Childhood: Investigating the Neurodevelopmental Gradient Hypothesis
Source: Int J Environ Res Public Health. 2021 Feb 10;18(4):1693. doi: 10.3390/ijerph18041693 (PMC7916459; doi:10.3390/ijerph18041693)
Supplement: Supplementary file 1 [file ijerph-18-01693-s001.pdf]

**Supplementary Table S1.** Performance among NDD children/adolescents as a whole group and controls

| | Area ($mm^2$) | | Perimeter (mm) | | APAV (mm/sec) | | LAV (mm/sec) | |
|---|---|---|---|---|---|---|---|---|
| **Conditions** | **Controls** | **NDD** | **Controls** | **NDD** | **Controls** | **NDD** | **Controls** | **NDD** |
| | **Median (IQR)** | | | | | | | |
| **SOT1-EO** | 30 (21-63) | 79 (46-143) | 123 (92-140) | 156 (120-194) | 3 (2-4) | 4 (3-5) | 2 (2-3) | 4 (3-4) |
| **SOT2-EC** | 58 (47-91) | 164 (78-285) | 157 (132-204) | 242 (167-290) | 4 (4-6) | 7 (5-9) | 3 (3-4) | 5 (3-6) |
| **SOT3-SV** | 66 (35-93) | 166 (96-275) | 150 (117-177) | 210 (168-288) | 4 (3-5) | 6 (5-8) | 3 (2-4) | 5 (3-6) |
| **SOT4-EOSS** | 62 (42-96) | 132 (92-223) | 173 (144-221) | 240 (182-297) | 5 (4-6) | 7 (5-8) | 4 (4-5) | 6 (4-7) |
| **SOT5-ECSS** | 189 (134-264) | 400 (238-562) | 299 (234-346) | 421 (302-505) | 8 (7-10) | 11 (9-15) | 7 (5-8) | 9 (7-12) |
| **SOT6-SVSS** | 190 (132-294) | 490 (702-299) | 299 (212-343) | 409 (301-510) | 8 (6-10) | 11 (8-14) | 7 (5-7) | 9 (7-12) |

NDD, Neurodevelopmental Disorders; APAV, Anterior-Posterior Average Velocity; LAV, Lateral Average Velocity; IQR, interquartile range; SOT, Sensory Organization Test; EO, Eyes Open; EC, Eyes Closed; SV, Sway-referenced Vision; EOSS, Eyes Open Sway-referenced Support; ECSS, Eyes Closed Sway-referenced Support; SVSS, Sway-referenced Vision Sway-referenced Support

**Supplementary Table S2.** Performance in the Area parameter among NDD and controls

| **Conditions** | **ASD** | **ADHD** | **TS** | **Controls** |
|---|---|---|---|---|
| | **Median (IQR)** | | | |
| **SOT1-EO** | 122.5 (78.5-191) | 68 (46-151) | 62.5 (38-147.5) | 30 (21-63) |
| **SOT2-EC** | 265.5 (103-395) | 170 (86-330) | 97 (66-250) | 58 (47-91) |
| **SOT3-SV** | 214 (141.5-302.5) | 178 (104-351) | 123 (56-241) | 66 (35-93) |
| **SOT4-EOSS** | 149 (95.5-296) | 142 (78-259) | 116.5 (97-187) | 62 (42-96) |
| **SOT5-ECSS** | 430 (313-712) | 469 (280-567) | 273 (202-487) | 189 (134-264) |
| **SOT6-SVSS** | 575 (297.5-708) | 552 (381-716) | 355.5 (252-640) | 190 (132-294) |

NDD, Neurodevelopmental Disorders; ASD, Autism Spectrum Disorder; ADHD, Attention Deficit Hyperactivity Disorder; TS, Tourette Syndrome; IQR, interquartile range; SOT, Sensory Organization Test; EO, Eyes Open; EC, Eyes Closed; SV, Sway-referenced Vision; EOSS, Eyes Open Sway-referenced Support; ECSS, Eyes Closed Sway-referenced Support; SVSS, Sway-referenced Vision Sway-referenced Support

**Supplementary Table S3.** Performance in the Perimeter parameter among NDD and controls

| **Conditions** | **ASD** | **ADHD** | **TS** | **Controls** |
|---|---|---|---|---|
| | **Median (IQR)** | | | |
| **SOT1-EO** | 182.5 (126.5-233) | 161 (120-196) | 140.5 (112-178) | 123 (92-140) |
| **SOT2-EC** | 288.5 (203-326.5) | 260 (172-336) | 217.5 (150-256) | 157 (132-204) |
| **SOT3-SV** | 238 (177-321.5) | 242 (181-303) | 181.5 (154-233) | 150 (117-177) |
| **SOT4-EOSS** | 243.5 (184-292.5) | 268 (193-360) | 232.5 (172-283) | 173 (144-221) |
| **SOT5-ECSS** | 428 (300.5-539.5) | 446 (326-568) | 343.5 (287-471) | 299 (234-346) |
| **SOT6-SVSS** | 425.5 (303.5-554) | 464 (337-530) | 346 (297-464) | 299 (212-343) |

NDD, Neurodevelopmental Disorders; ASD, Autism Spectrum Disorder; ADHD, Attention Deficit Hyperactivity Disorder; TS, Tourette Syndrome; IQR, interquartile range; SOT, Sensory Organization Test; EO, Eyes Open; EC, Eyes

Closed; SV, Sway-referenced Vision; EOSS, Eyes Open Sway-referenced Support; ECSS, Eyes Closed Sway-referenced Support; SVSS, Sway-referenced Vision Sway-referenced Support

**Supplementary Table S4.** Performance in the APAV parameter among NDD and controls

| Conditions | ASD | ADHD | TS | Healthy |
|---|---|---|---|---|
| | | Median (IQR) | | |
| **SOT1-EO** | 5 (3.5-6.5) | 4 (3-5) | 4 (3-5) | 3 (2-4) |
| **SOT2-EC** | 9 (5-9.5) | 7 (5-10) | 6.5 (4-7) | 4 (4-6) |
| **SOT3-SV** | 6.5 (5-9) | 7 (5-8) | 5 (4-7) | 4 (3-5) |
| **SOT4-EOSS** | 6.5 (5.5-7.5) | 8 (6-10) | 6 (4-8) | 5 (4-6) |
| **SOT5-ECSS** | 12 (8-16) | 12 (9-15) | 10 (9-13) | 8 (7-10) |
| **SOT6-SVSS** | 11 (8.5-15.5) | 12 (10-15) | 9.5 (8-13) | 8 (6-10) |

APAV, Anterior-Posterior Average Velocity; NDD, Neurodevelopmental Disorders; ASD, Autism Spectrum Disorder; ADHD, Attention Deficit Hyperactivity Disorder; TS, Tourette Syndrome; IQR, interquartile range; SOT, Sensory Organization Test; EO, Eyes Open; EC, Eyes Closed; SV, Sway-referenced Vision; EOSS, Eyes Open Sway-referenced Support; ECSS, Eyes Closed Sway-referenced Support; SVSS, Sway-referenced Vision Sway-referenced Support

**Supplementary Table S5.** Performance in the LAV parameter among NDD and controls

| Conditions | ASD | ADHD | TS | Healthy |
|---|---|---|---|---|
| | | Median (IQR) | | |
| **SOT1-EO** | 4(3-5) | 3(3-4) | 3(3-4) | 2(2-3) |
| **SOT2-EC** | 6(4.5-6.5) | 6(3-7) | 4(3-5) | 3(3-4) |
| **SOT3-SV** | 5(4-6.5) | 5(3-6) | 4(3-5) | 3(2-4) |
| **SOT4-EOSS** | 6(4-7) | 6(4-8) | 5.5(4-6) | 4(4-5) |
| **SOT5-ECSS** | 10(7-12) | 10(8-12) | 8(6-11) | 7(5-8) |
| **SOT6-SVSS** | 10(7-12.5) | 10(7-13) | 8(7-11) | 7(5-7) |

LAV, Lateral Average Velocity; NDD, Neurodevelopmental Disorders; ASD, Autism Spectrum Disorder; ADHD, Attention Deficit Hyperactivity Disorder; TS, Tourette Syndrome; IQR, interquartile range; SOT, Sensory Organization Test; EO, Eyes Open; EC, Eyes Closed; SV, Sway-referenced Vision; EOSS, Eyes Open Sway-referenced Support; ECSS, Eyes Closed Sway-referenced Support; SVSS, Sway-referenced Vision Sway-referenced Support

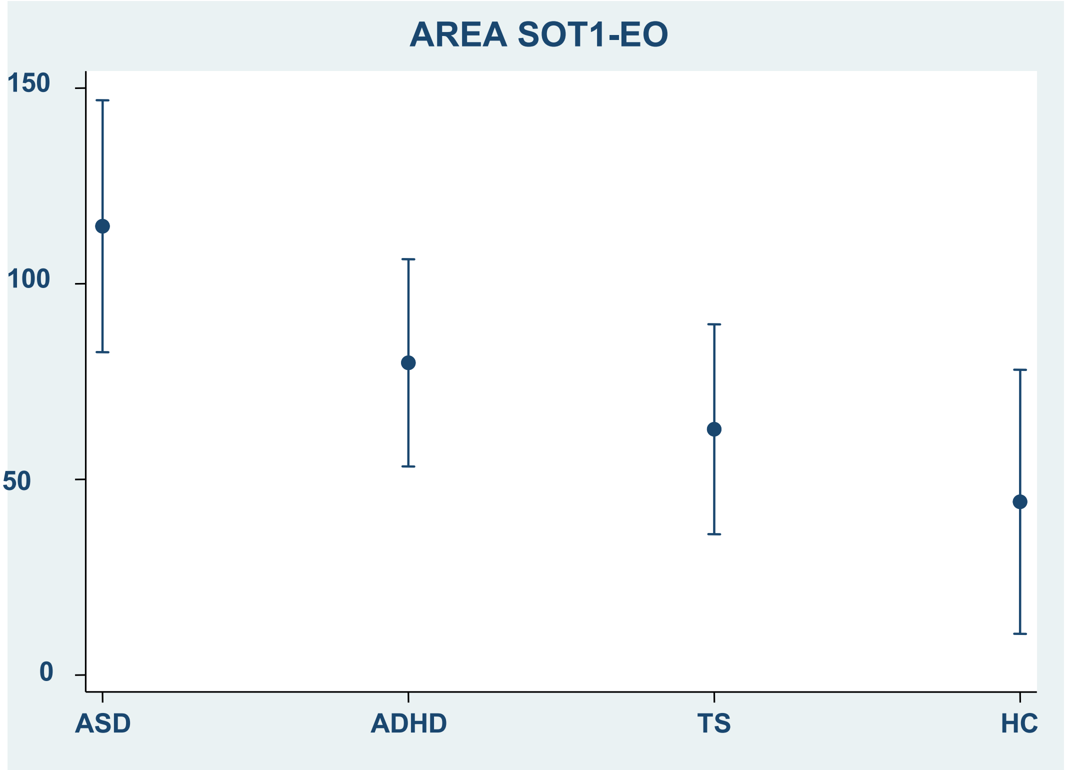
AREA SOT1-EO
150
100
50
0
ASD
ADHD
TS
HC
(a)

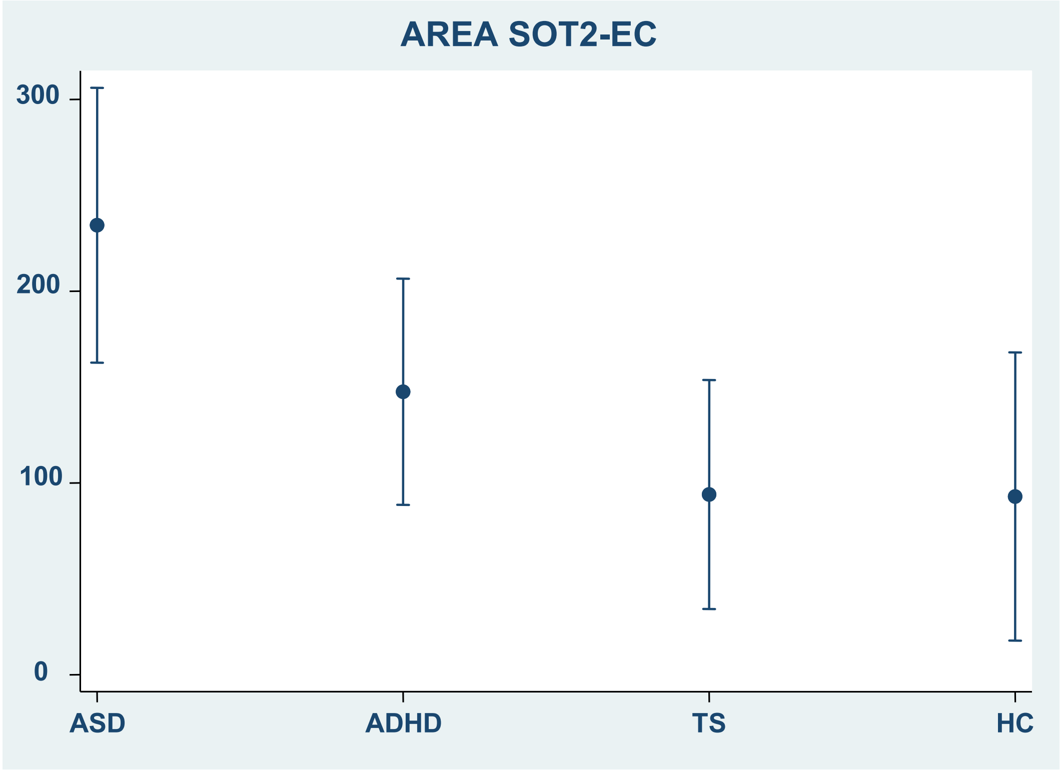
AREA SOT2-EC
300
200
100
0
ASD
ADHD
TS
HC
(b)

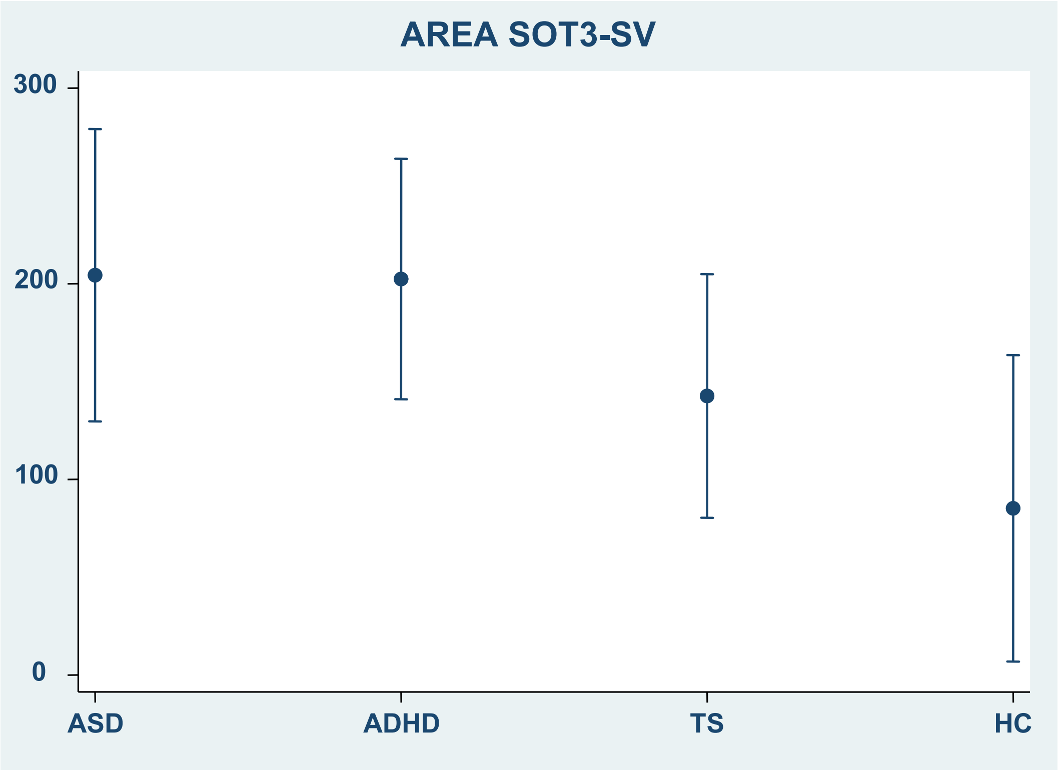
AREA SOT3-SV
300
200
100
0
ASD
ADHD
TS
HC

(c)

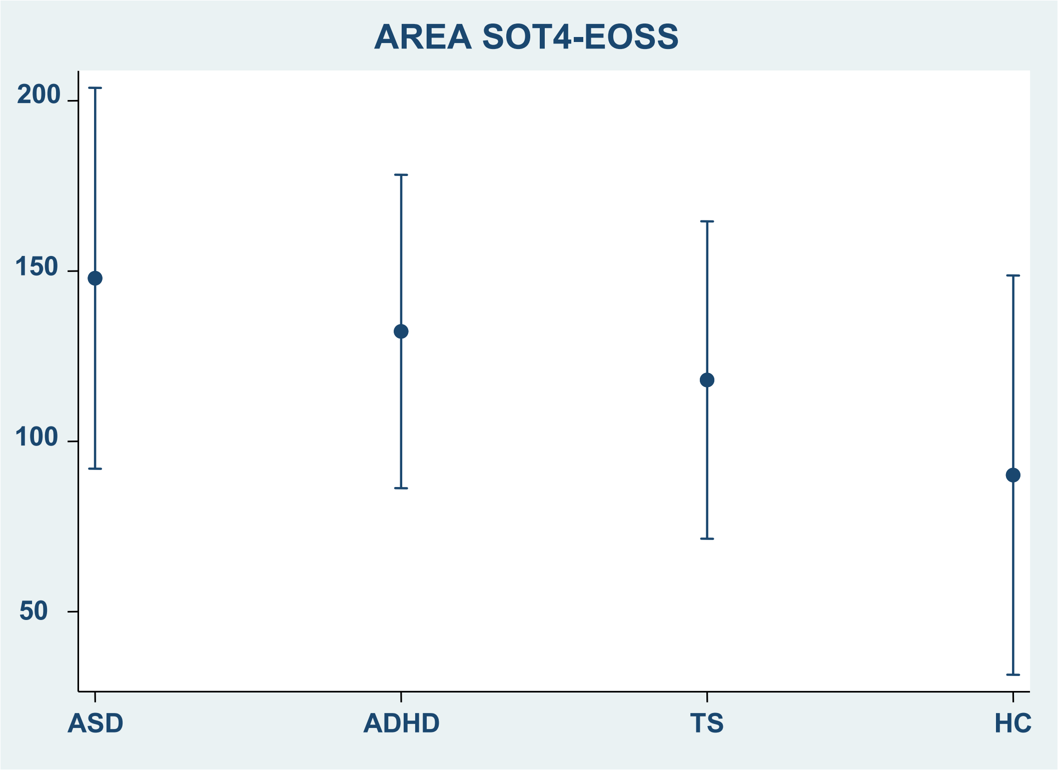
AREA SOT4-EOSS
200
150
100
50
ASD
ADHD
TS
HC

(d)

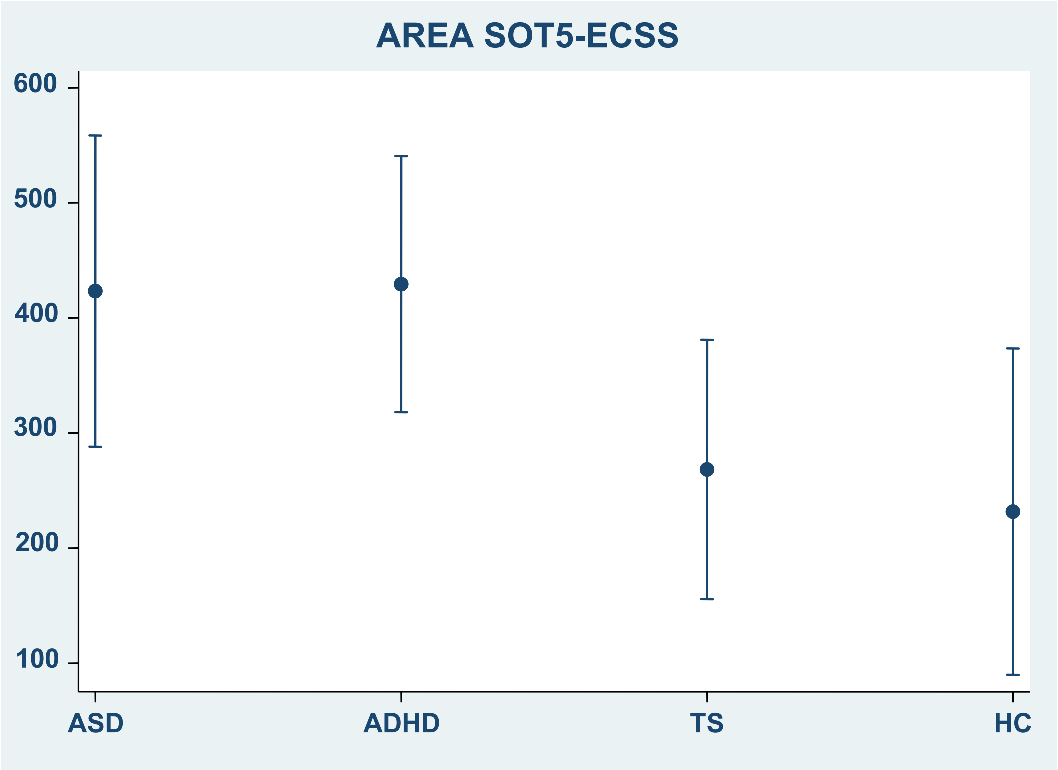


(**e**)

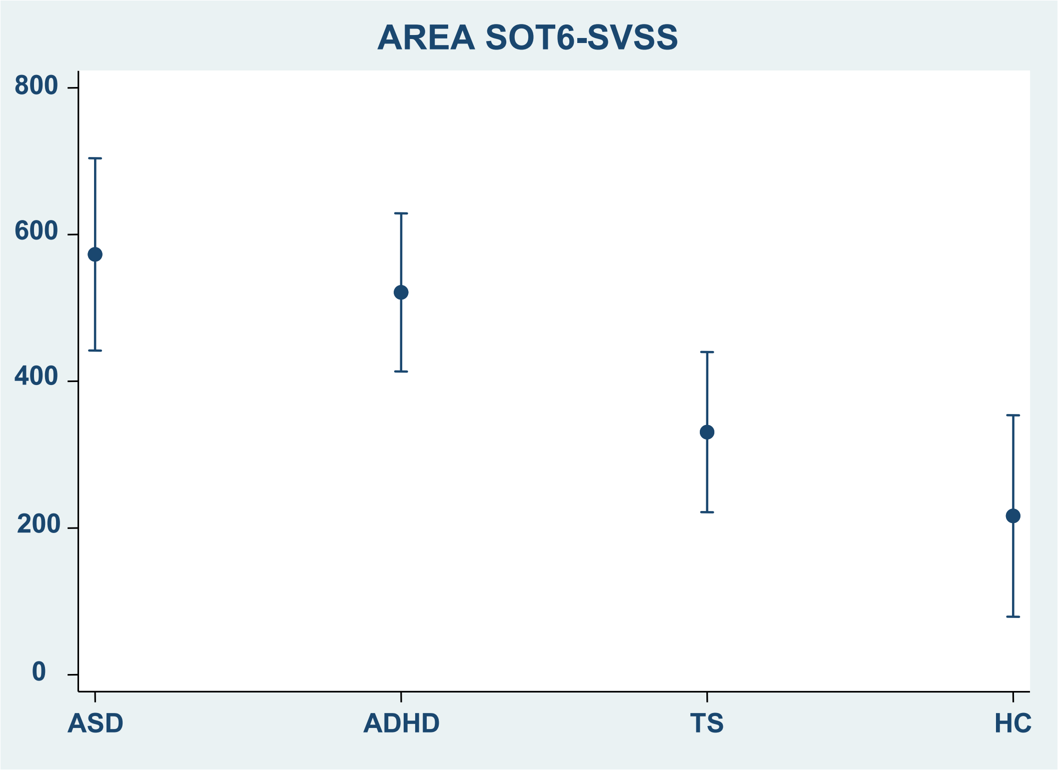


(**f**)

**Supplementary Figure S1**. The figure shows performance in the Area parameter among neurodevelopmental disorders and controls and bars show medians adjusted for gender and age. **(a)** SOT, Sensory Organization Test; EO, Eyes Open; **(b)** EC, Eyes Closed; **(c)** SV, Sway-referenced Vision; **(d)** EOSS, Eyes Open Sway-referenced Support; **(e)** ECSS, Eyes Closed Sway-referenced Support; **(f)** SVSS, Sway-referenced Vision Sway-referenced Support; ASD, Autism Spectrum Disorder; ADHD, Attention Deficit Hyperactivity Disorder; TS, Tourette Syndrome; HC, Healthy Controls.

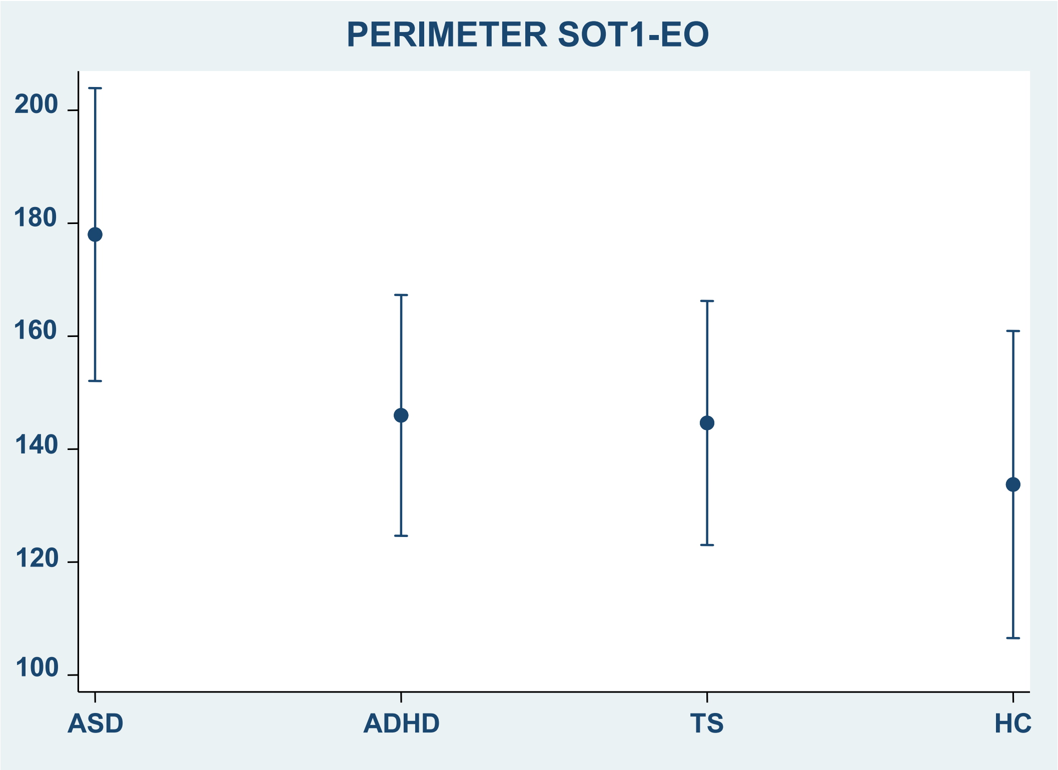
PERIMETER SOT1-EO
200
180
160
140
120
100
ASD
ADHD
TS
HC
(a)

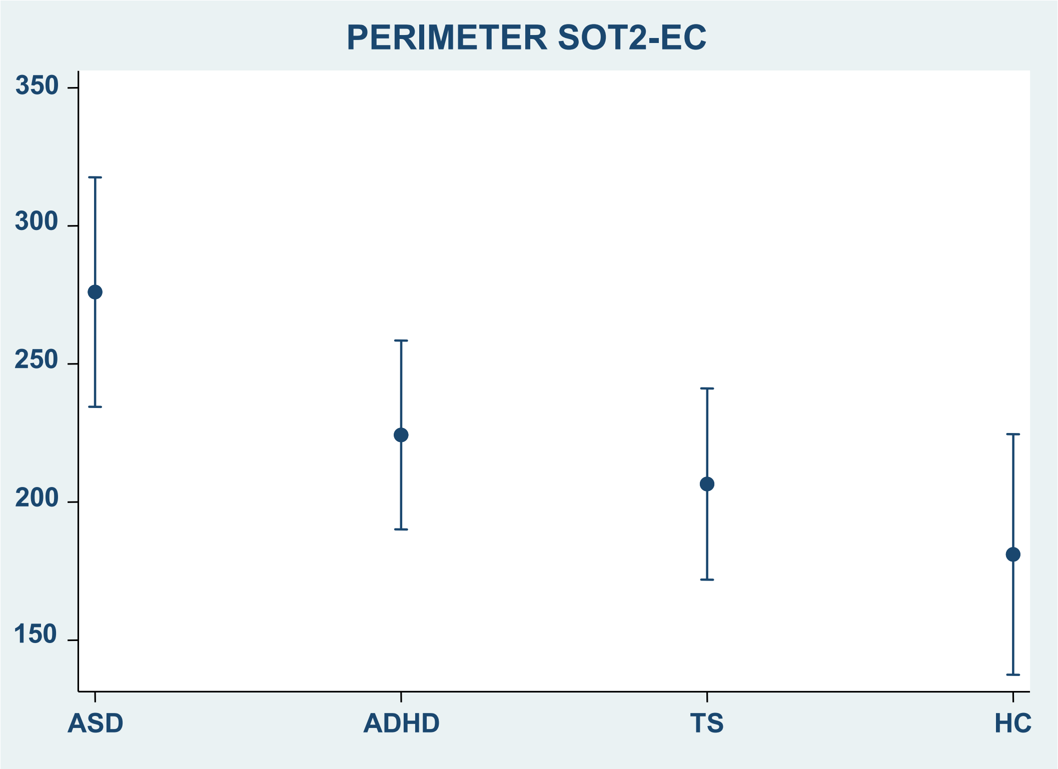
PERIMETER SOT2-EC
350
300
250
200
150
ASD
ADHD
TS
HC
(b)

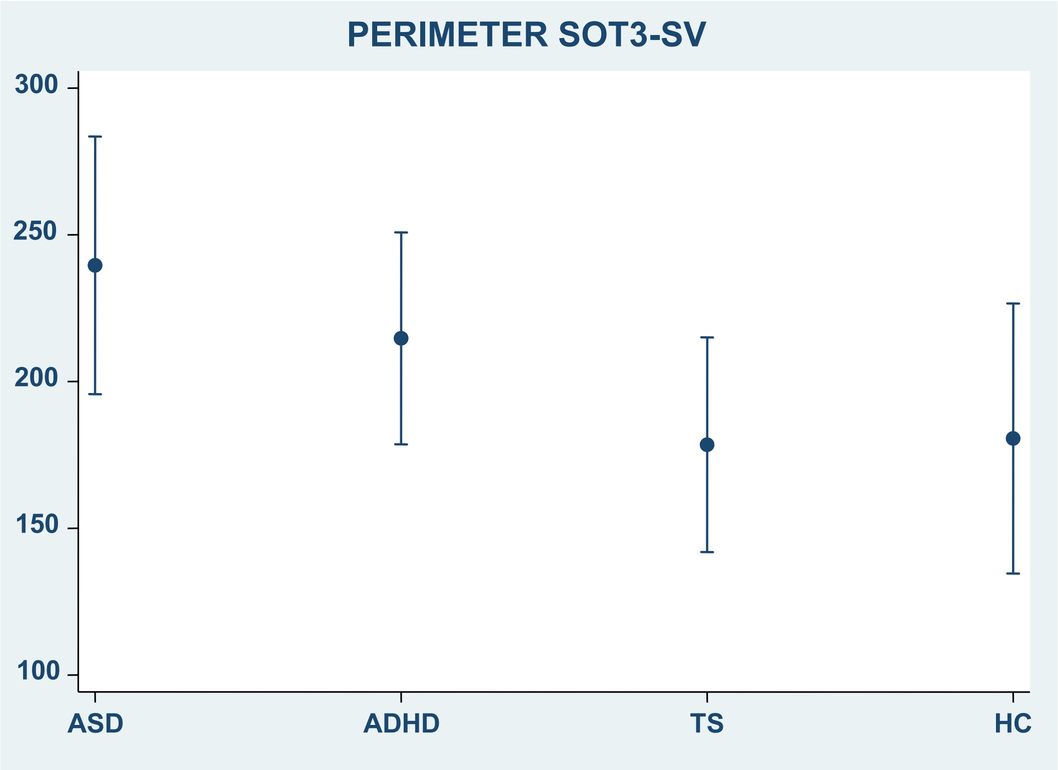
PERIMETER SOT3-SV
300
250
200
150
100
ASD
ADHD
TS
HC
(c)

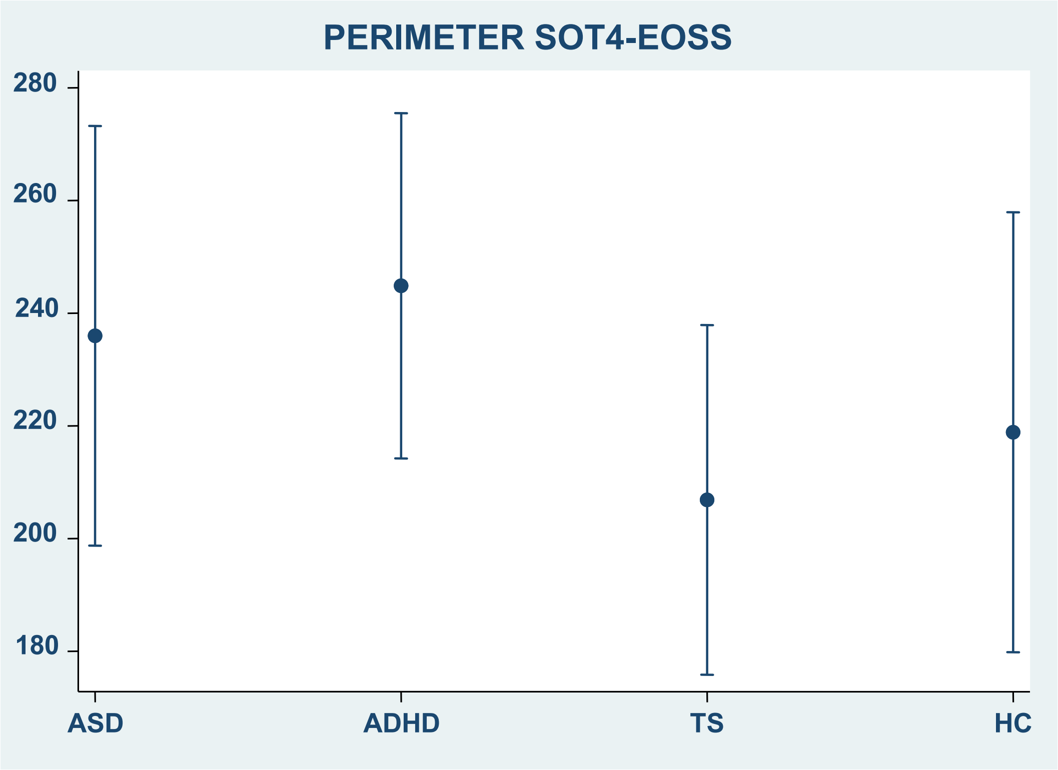
PERIMETER SOT4-EOSS
280
260
240
220
200
180
ASD
ADHD
TS
HC
(d)

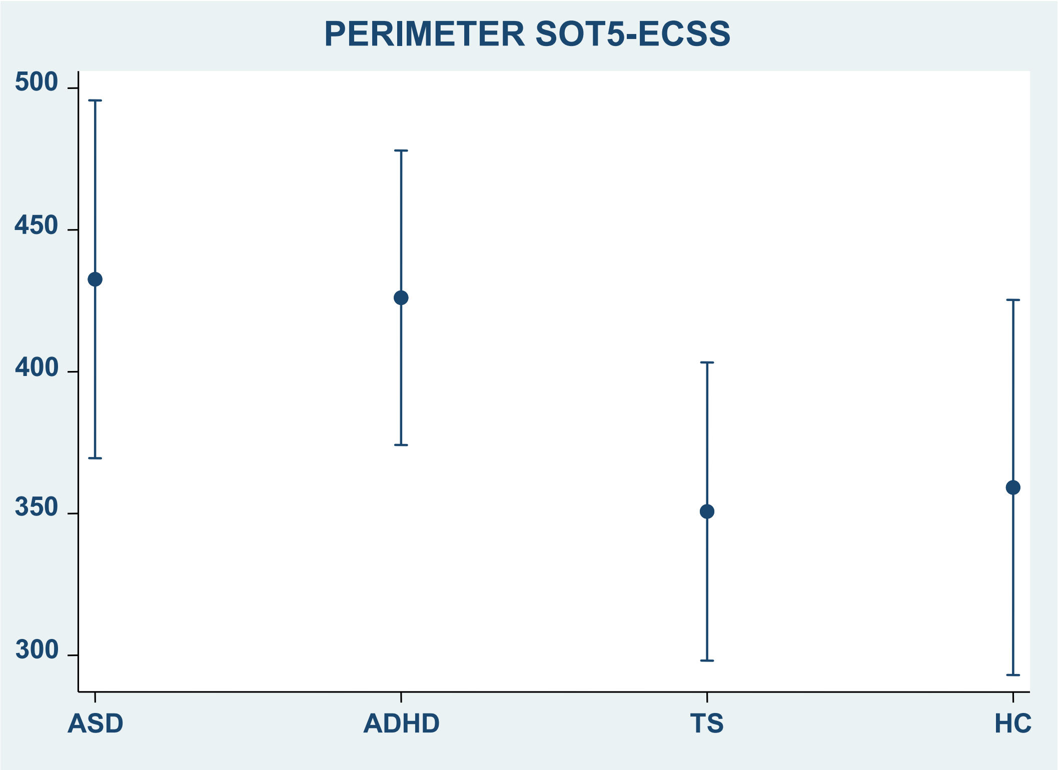


(e)

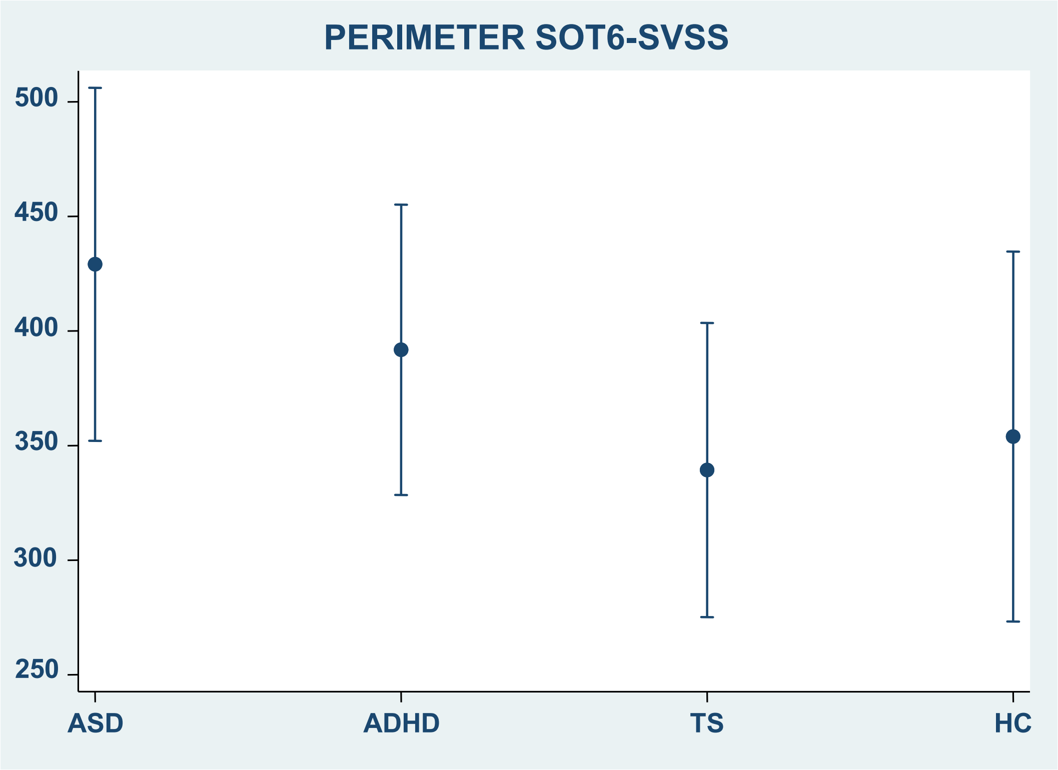


(f)

**Supplementary Figure S2**. The figure shows performance in the Perimeter parameter among neurodevelopmental disorders and controls and bars show medians adjusted for gender and age. **(a)** SOT, Sensory Organization Test; EO, Eyes Open; **(b)** EC, Eyes Closed; **(c)** SV, Sway-referenced Vision; **(d)** EOSS, Eyes Open Sway-referenced Support; **(e)** ECSS, Eyes Closed Sway-referenced Support; **(f)** SVSS, Sway-referenced Vision Sway-referenced Support; ASD, Autism Spectrum Disorder; ADHD, Attention Deficit Hyperactivity Disorder; TS, Tourette Syndrome; HC, Healthy Controls.

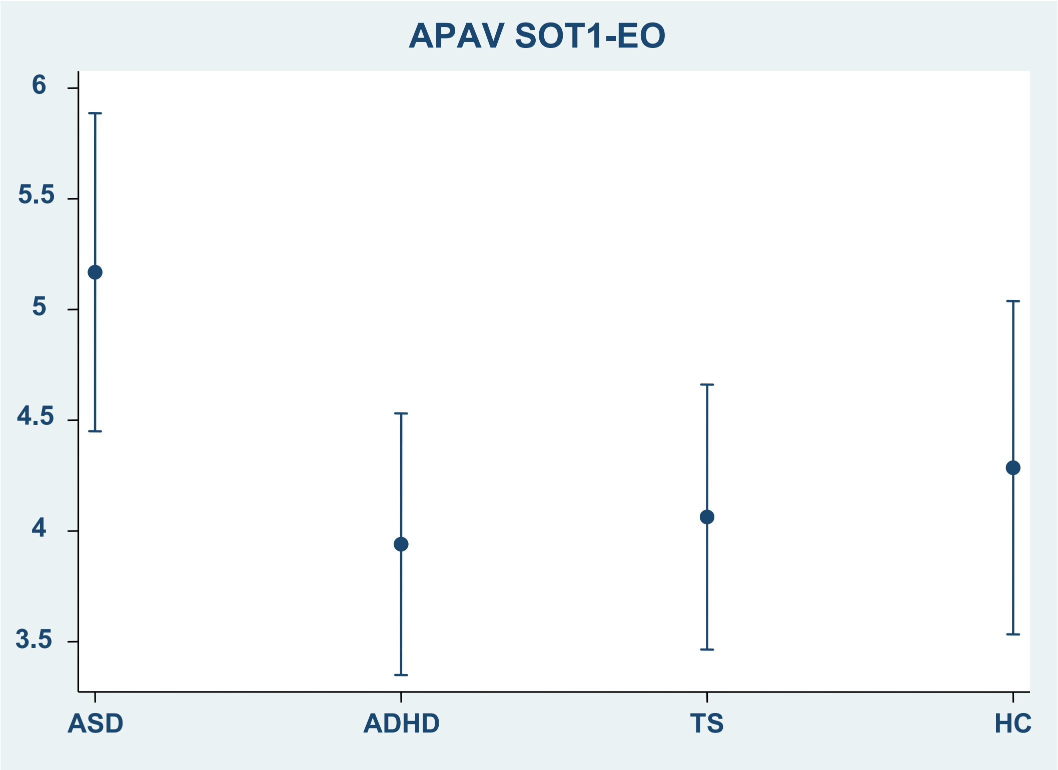
APAV SOT1-EO
6
5.5
5
4.5
4
3.5
ASD
ADHD
TS
HC

(a)

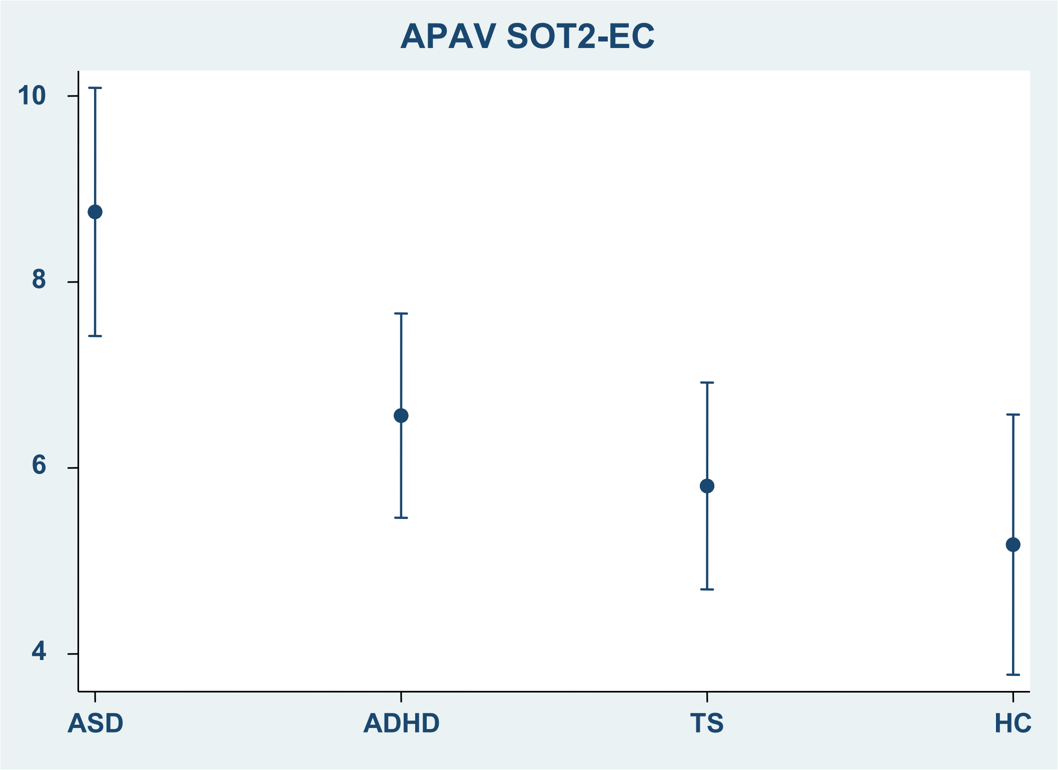
APAV SOT2-EC
10
8
6
4
ASD
ADHD
TS
HC

(b)

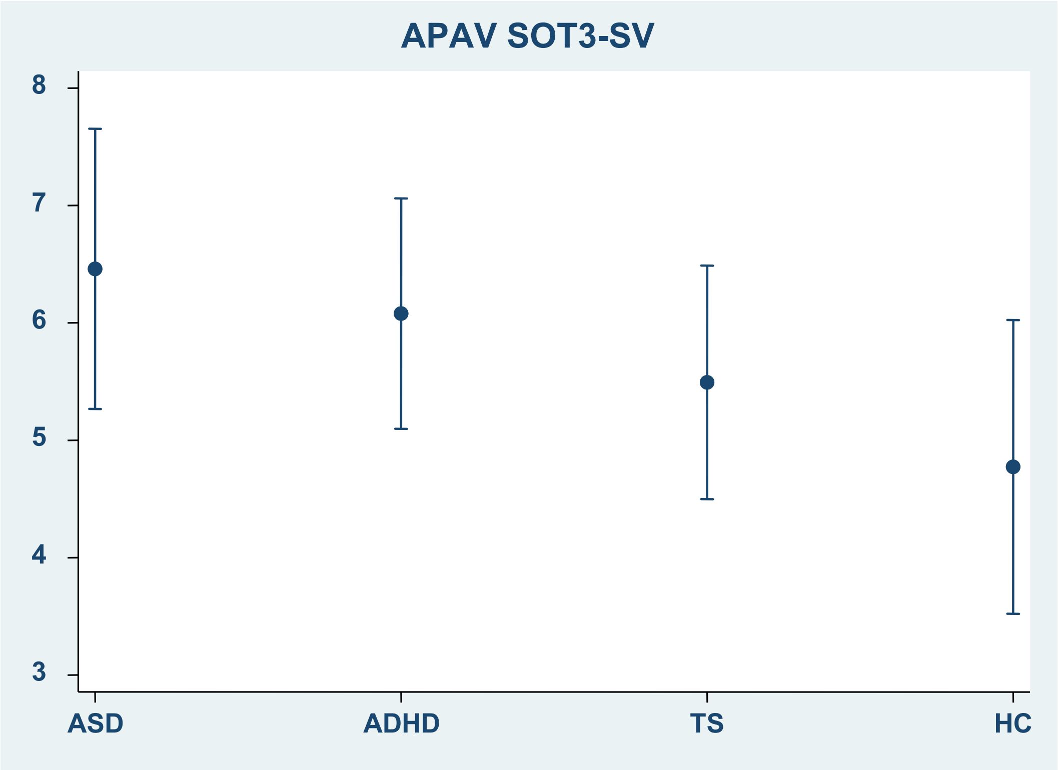
APAV SOT3-SV
8
7
6
5
4
3
ASD
ADHD
TS
HC
(c)

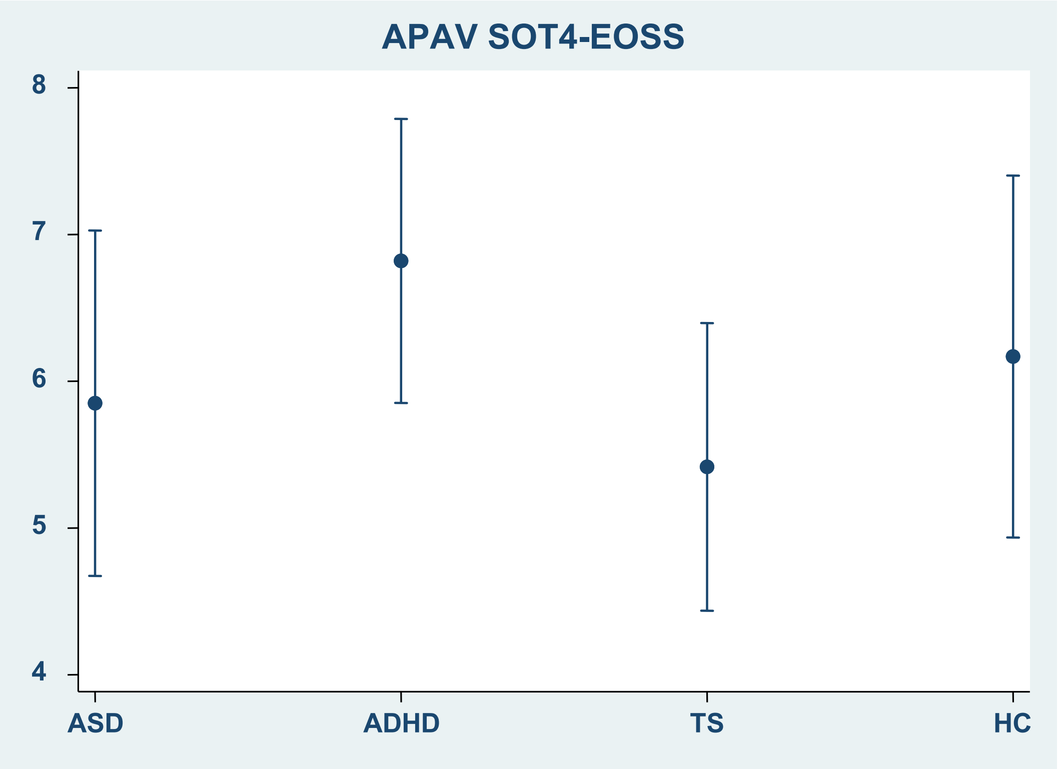
APAV SOT4-EOSS
8
7
6
5
4
ASD
ADHD
TS
HC
(d)

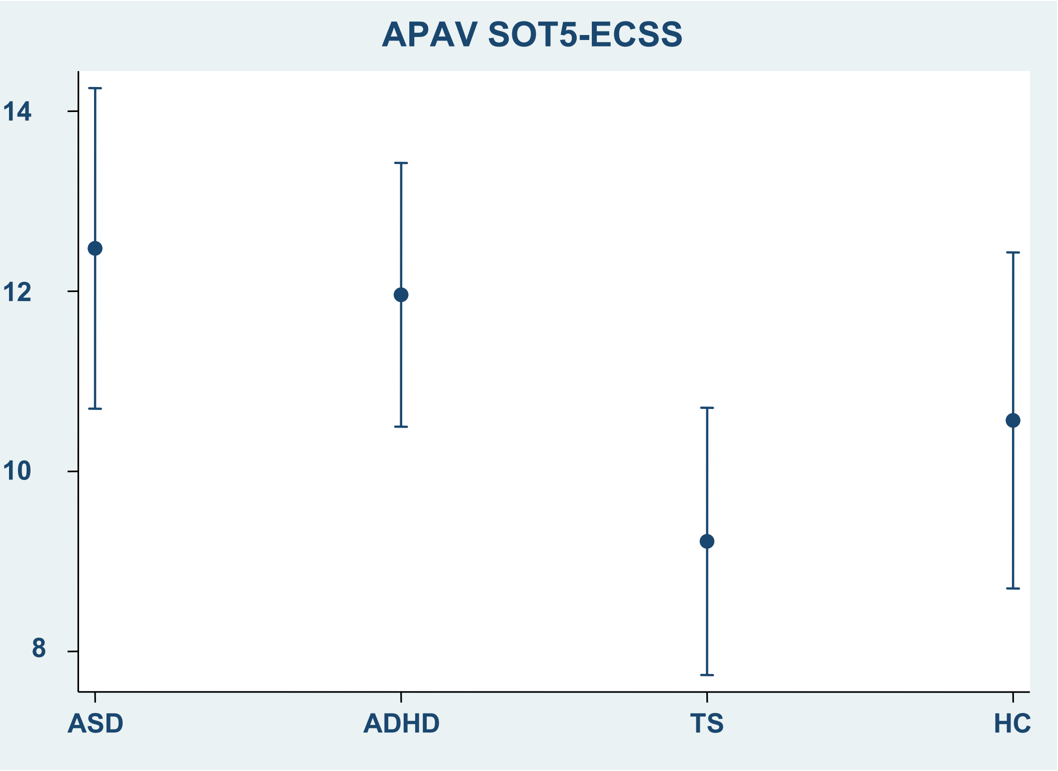


(**e**)

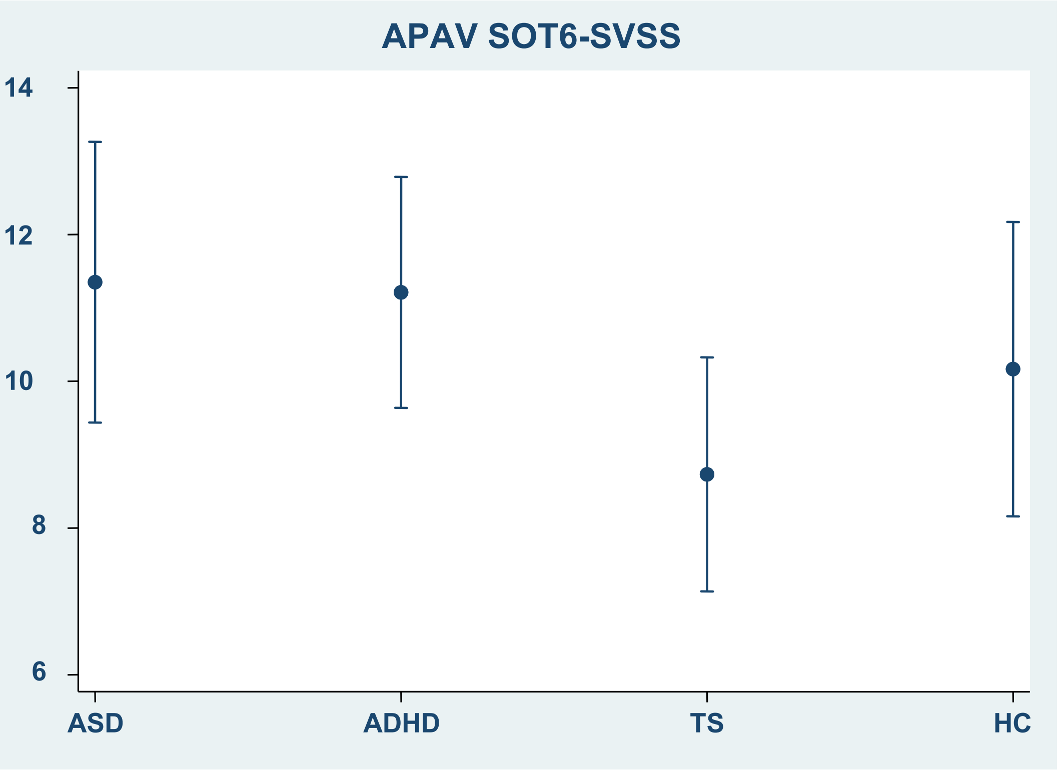


(**f**)

**Supplementary Figure S3**. The figure shows performance in the Anterior-Posterior Average Velocity parameter among neurodevelopmental disorders and controls and bars show medians adjusted for gender and age. **(a)** APAV, Anterior-Posterior Average Velocity; SOT, Sensory Organization Test; EO, Eyes Open; **(b)** EC, Eyes Closed; **(c)** SV, Sway-referenced Vision; **(d)** EOSS, Eyes Open Sway-referenced Support; **(e)** ECSS, Eyes Closed Sway-referenced Support; **(f)** SVSS, Sway-referenced Vision Sway-referenced Support; ASD, Autism Spectrum Disorder; ADHD, Attention Deficit Hyperactivity Disorder; TS, Tourette Syndrome; HC, Healthy Controls.

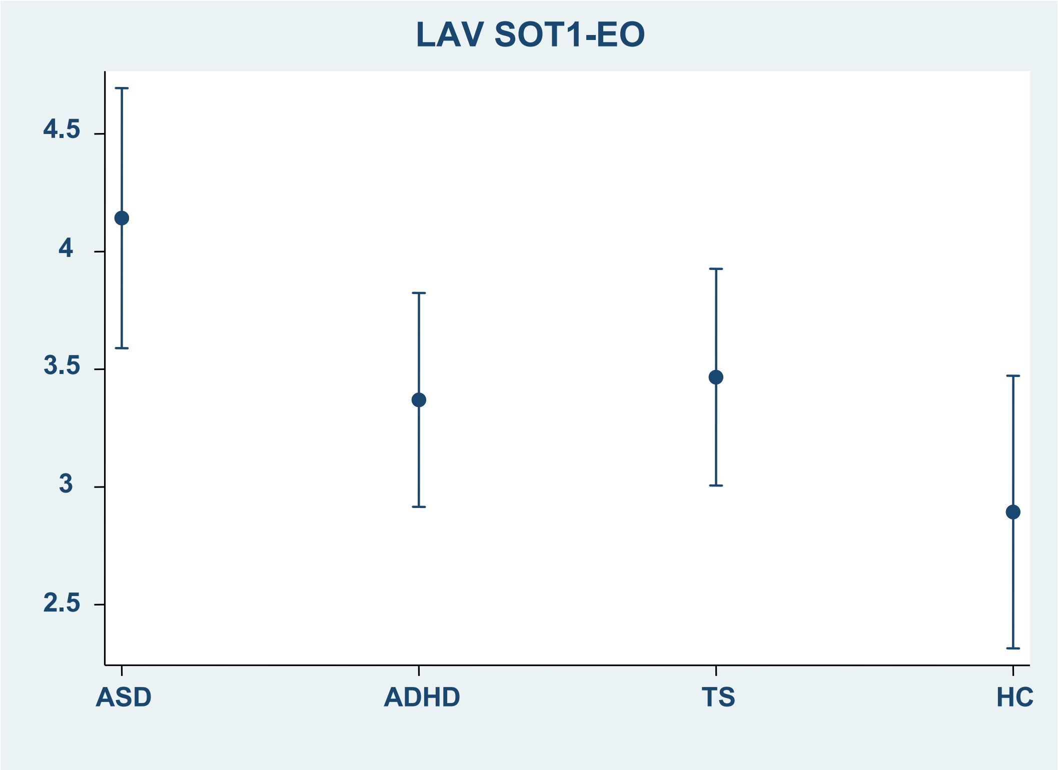
LAV SOT1-EO
4.5
4
3.5
3
2.5
ASD
ADHD
TS
HC
(a)

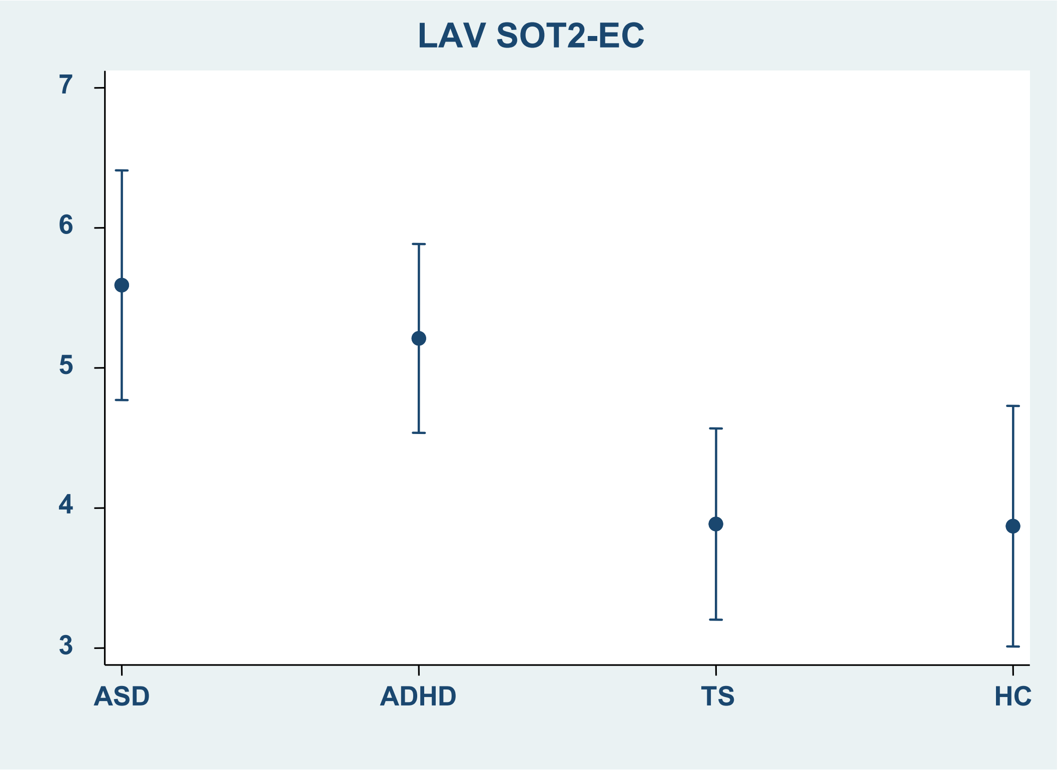
LAV SOT2-EC
7
6
5
4
3
ASD
ADHD
TS
HC
(b)

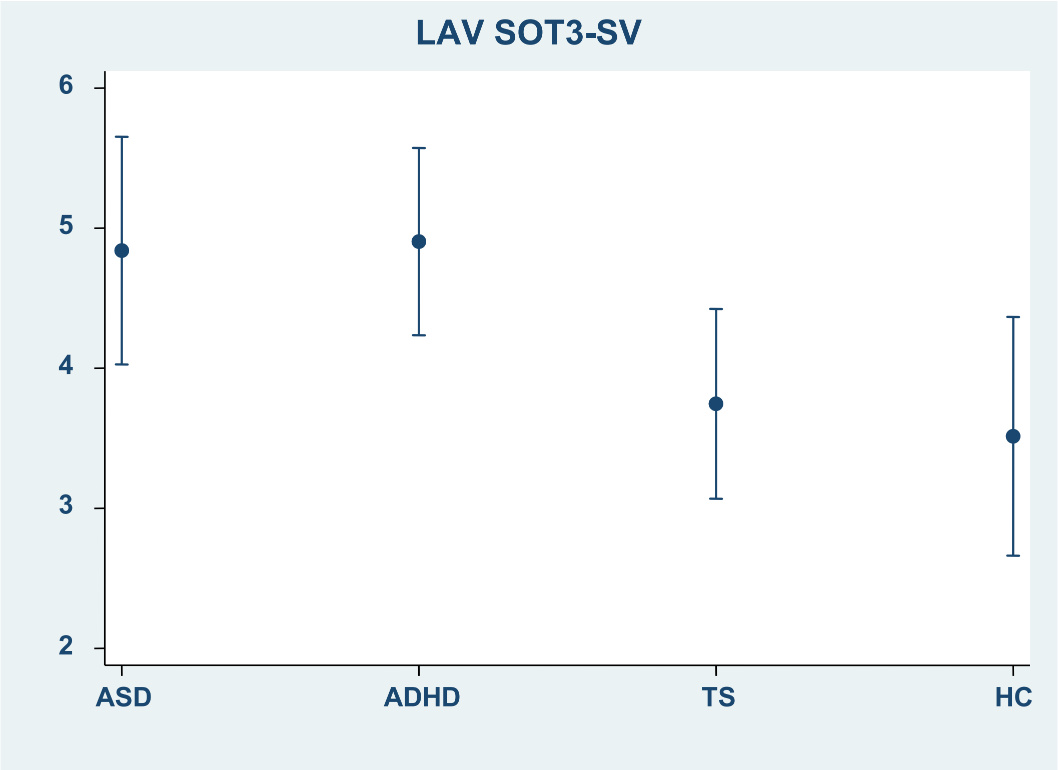
LAV SOT3-SV
6
5
4
3
2
ASD
ADHD
TS
HC
(c)

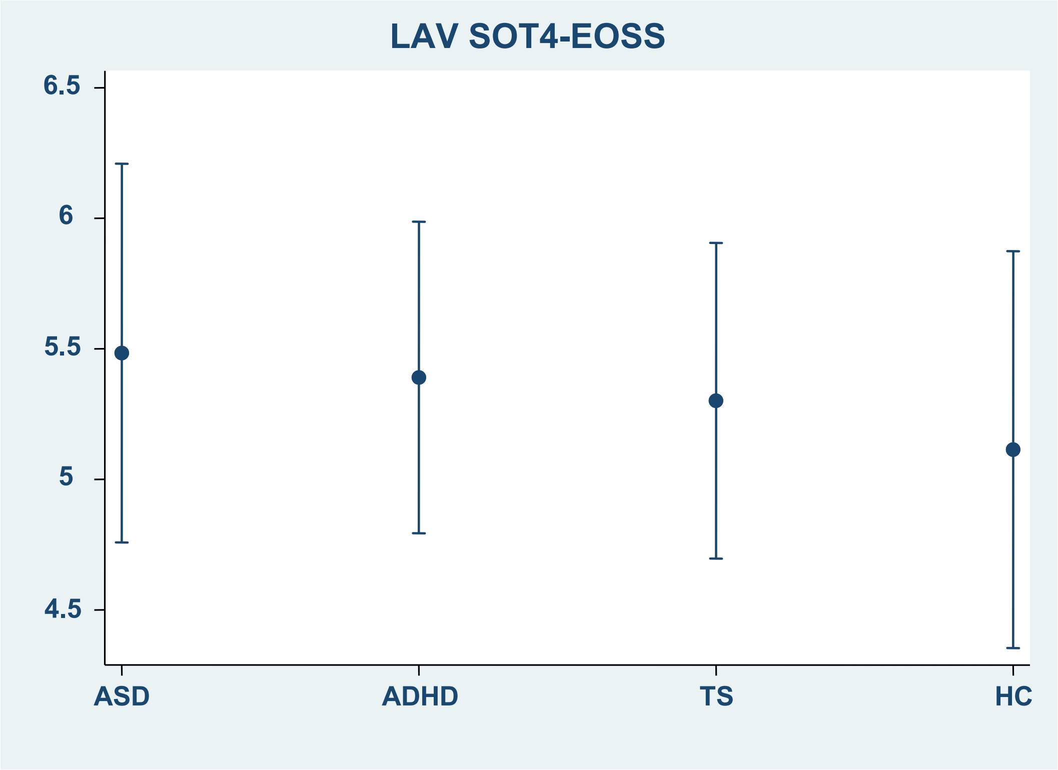
LAV SOT4-EOSS
6.5
6
5.5
5
4.5
ASD
ADHD
TS
HC
(d)

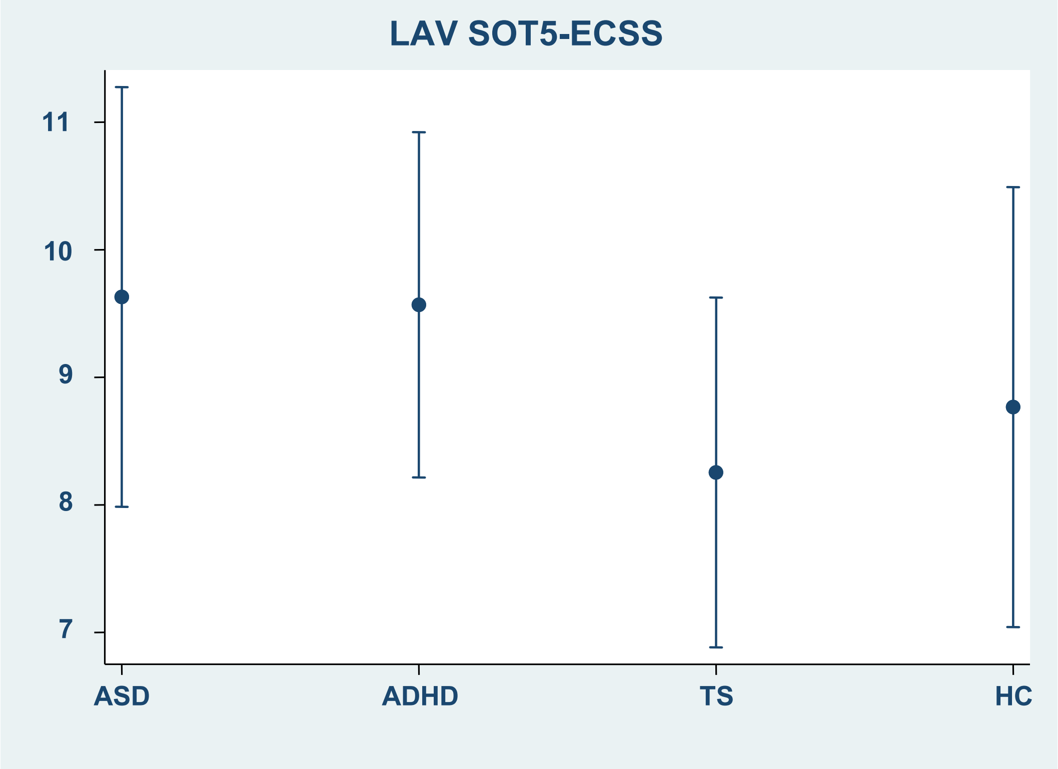


(**e**)

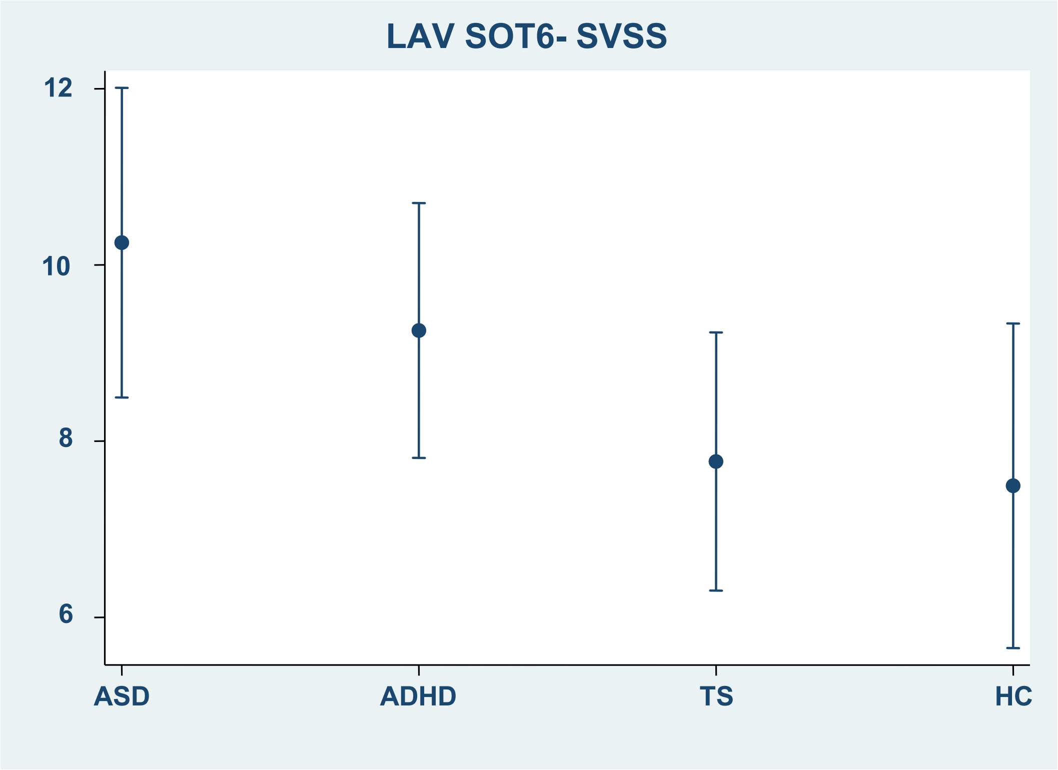


(**f**)

**Supplementary Figure S4**. The figure shows performance in the Lateral Average Velocity parameter among neurodevelopmental disorders and controls and bars show medians adjusted for gender and age. **(a)** LAV, Lateral Average Velocity; SOT, Sensory Organization Test; EO, Eyes Open; **(b)** EC, Eyes Closed; **(c)** SV, Sway-referenced Vision; **(d)** EOSS, Eyes Open Sway-referenced Support; **(e)** ECSS, Eyes Closed Sway-referenced Support; **(f)** SVSS, Sway-referenced Vision Sway-referenced Support; ASD, Autism Spectrum Disorder; ADHD, Attention Deficit Hyperactivity Disorder; TS, Tourette Syndrome; HC, Healthy Controls.
